# Extracellular bimolecular fluorescence complementation for investigating membrane protein dimerization: a proof of concept using class B GPCRs

**DOI:** 10.1042/BSR20240449

**Published:** 2024-10-23

**Authors:** Michael L. Garelja, Tyla I. Alexander, Christopher S. Walker, Debbie L. Hay

**Affiliations:** 1 Department of Pharmacology and Toxicology, University of Otago, Dunedin, 9016, New Zealand; 2 School of Biological Sciences, University of Auckland, Auckland, 1010, New Zealand; 3 Maurice Wilkins Centre for Molecular Biodiscovery, University of Auckland, Auckland, New Zealand

**Keywords:** Bimolecular Fluorescence Complementation, Calcitonin gene-related peptide, calcitonin receptor, dimerization, g protein-coupled receptors, receptor activity-modifying protein

## Abstract

Bimolecular fluorescence complementation (BiFC) methodology uses split fluorescent proteins to detect interactions between proteins in living cells. To date, BiFC has been used to investigate receptor dimerization by splitting the fluorescent protein between the intracellular portions of different receptor components. We reasoned that attaching these split proteins to the extracellular N-terminus instead may improve the flexibility of this methodology and reduce the likelihood of impaired intracellular signal transduction. As a proof-of-concept, we used receptors for calcitonin gene-related peptide, which comprise heterodimers of either the calcitonin or calcitonin receptor-like receptor in complex with an accessory protein (receptor activity-modifying protein 1). We created fusion constructs in which split mVenus fragments were attached to either the C-termini or N-termini of receptor subunits. The resulting constructs were transfected into Cos7 and HEK293S cells, where we measured cAMP production in response to ligand stimulation, cell surface expression of receptor complexes, and BiFC fluorescence. Additionally, we investigated ligand-dependent internalization in HEK293S cells. We found N-terminal fusions were better tolerated with regards to cAMP signaling and receptor internalization. N-terminal fusions also allowed reconstitution of functional fluorescent mVenus proteins; however, fluorescence yields were lower than with C-terminal fusion. Our results suggest that BiFC methodologies can be applied to the receptor N-terminus, thereby increasing the flexibility of this approach, and enabling further insights into receptor dimerization.

## Introduction

Bimolecular fluorescence complementation (BiFC) is a fluorescence-based technique that is used to investigate interactions between proteins [1]. The technique involves splitting a fluorescent protein (such as mVenus) into two halves, creating an N-terminal portion (V_1–154_) and a C-terminal portion (V_155–238_). These halves do not fluoresce on their own, but when in close proximity, they can refold to create a fully functional fluorescent protein. This phenomenon is exploited in BiFC methodologies by fusing the fluorescent protein fragments to distinct proteins. If the target proteins are in close proximity, this facilitates the formation of a fluorescent protein, and thus the generation of a fluorescent signal [1].

BiFC can be used to explore the dimerization of membrane proteins, such as dimers of G protein-coupled receptors (GPCRs), heterodimers of GPCRs and accessory proteins, or of receptor tyrosine kinase (RTK) subunits. These interactions have all been probed with the split fluorescent proteins fused to the intracellular portion of the protein [2–5]. Intracellular fusions may interfere with the signaling and post-stimulation trafficking of receptors by altering the ability of intracellular effectors to bind to or interact with the receptor [6,7]. To our knowledge, extracellular fusion of split fluorescent proteins has not been formally trialed using BiFC [2]. We posited that N-terminal fusion may be better tolerated with regards to receptor function. N-terminal fusion of large proteins to a GPCR is not without precedent; NanoBit ligand binding studies that incorporate a similar sized fusion protein attached the receptor N-terminus are able to bind ligands, and fusion constructs which attach NanoLuc and SNAP fusion tags to the extracellular portion of the receptor can detect dimers formed between receptors and accessory proteins [8–10]. Thus, we investigated whether an N-terminal fusion approach could be extrapolated to BiFC methodology.

We used receptors for calcitonin gene-related peptide (CGRP) as a model system. Functional CGRP-responsive receptors are heterodimers comprising a class B GPCR (either the calcitonin receptor [CTR] or the calcitonin receptor-like receptor [CLR]) in complex with receptor activity-modifying protein 1 (RAMP1). These proteins have large extracellular N-termini, and thus in addition to being models of class B GCPRs they also make good models for other GPCRs that have large N-termini (e.g. Class C and Frizzled receptors), as well as non-GPCR receptors which have large extracellular domains, such as RTKs. We used these CGRP-responsive receptors as a model system because heterodimerization of CLR or CTR with RAMP1 is well supported [11–14]. In the absence of RAMP co-expression, CLR is trapped intracellularly and unable to respond to peptide stimulation. Co-expression with RAMP1 facilitates the trafficking of CLR to the cell surface where the CLR/RAMP1 complex acts as a high affinity receptor for CGRP; this complex is the canonical CGRP receptor [11]. Although CTR traffics to the cell surface and functions as a receptor in the absence of RAMP co-expression, CGRP is a weak agonist at CTR alone. In contrast, CGRP is a high-potency agonist of CTR/RAMP1 (known as the amylin [AMY]_1_ receptor) [11]. Thus, these two CGRP-responsive receptors are dimers (one obligate and one not), making them a useful model for investigating new tools that study dimerization.

Previously, BiFC methodology has been used to study the canonical CGRP receptor [15]. In this original study, enhanced yellow fluorescent protein (eYFP) was split and fused to the C-termini of CLR and RAMP1. When co-expressed, these constructs created a receptor that was able to recruit β-arrestin in response to αCGRP stimulation, and a functional eYFP protein which could be detected by fluorescence microscopy. However, there was no investigation into whether BiFC fusion influenced the cell surface expression of the receptor complex or interfered with signaling events distinct from β-arrestin recruitment such as cell signaling or receptor internalization [15]. C-terminal BiFC methodology has also been used to investigate complex formation between RAMPs and the secretin receptor [16].

In the present study, we created and tested a series of constructs in which split mVenus fragments were fused to either the C-termini or N-termini of CTR, CLR, and RAMP1 to compare the positional effect of fluorescent protein fragment placement. These fusion constructs were investigated for their ability to produce cAMP in response to CGRP stimulation, to traffic to the cell surface, and to form fluorescent mVenus proteins. We also investigated whether fusion orientation influenced the regulation of the CLR/RAMP1 complexes following ligand stimulation; the CTR/RAMP1 complex was not investigated as it does not show ligand-dependent internalization [17,18].

## Materials and methods

### Materials

Human αCGRP was synthesized in-house using an Fmoc solid-phase synthesis approach as described previously [19]. Human calcitonin was purchased commercially (Cat# 4014409, Bachem, Bubendorf, Switzerland). Cy5-labelled CGRP (Cy5 label on position 3) was synthesized in-house as previously described [17].

### Expression constructs

Human CLR and CTR with N-terminal hemagglutinin (HA) epitope tags were used in these experiments. The CTR used throughout this study was the most common CT_(a)_ splice variant with the Leu447 polymorphism. Human RAMP1 with an N-terminal myc epitope tag was also used in these experiments. These constructs are referred to as wild type (WT) throughout the manuscript. They were well-validated in previous studies and have been shown to have comparable pharmacology to untagged receptor constructs [13,20,21]. To simplify the nomenclature, these epitope tags are omitted from the descriptions of the receptors. Hence, CLR and CTR refer to HA-CLR and HA-CTR, respectively, while RAMP1 refers to myc-RAMP1. These constructs were used as a starting point for all modifications. All constructs were encoded in pcDNA 3.1; pcDNA 3.1 alone was also used as a vector control throughout the present study. For full sequences of WT and modified receptors, see supplementary information.

### Insert design

The mVenus halves used in the present study were as described by Kodama *et al*. mVenus was preferred over eYFP due to its reduced self-assembly, higher fluorescent yield, and its ability to mature at 37°C [22]. The halves were V_1–154_, consisting of mVenus residues 1–154 (including the I152L mutation which reduces self-assembly) and V_155–238_, consisting of mVenus residues 155–238. A flexible linker sequence was used to attach V_1–154_ or V_155–238_ to the receptor subunits to facilitate proper protein folding and maximize the bioactivity, we refer to this sequence as the Venus linker [23]. When conjugated to the intracellular C-terminus of receptor subunits, the Venus linker was attached to the N-terminus of the mVenus halves, while when conjugated to the extracellular N-terminus of receptor subunits, the Venus linker was attached to the C-terminus of the mVenus halves (see Supplemental Information pages 3–8 for further information).

**Figure 1:**
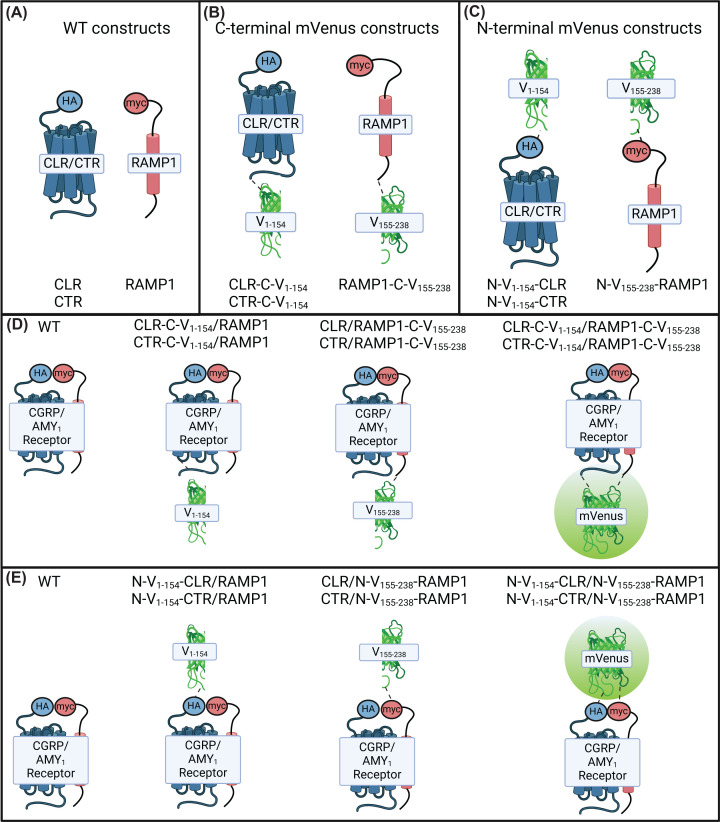
Fusion constructs and combinations for BiFC receptor testing Schematic detailing the fusion constructs created and the combinations tested. (**A**) WT receptor constructs. (**B**) constructs in which the BiFC components have been fused to the intracellular C-termini of receptors. (**C**) constructs with the BiFC components fused to the extracellular N-termini of receptors. (**D,E**) outline the combinations of constructs tested and provide the naming convention used to refer to them throughout the paper. Images made using biorender.com.

For fusion constructs in which the BiFC fragments were fused to the C-termini of receptors, we utilized the additional amino acid sequence “VPVNSGGGGS” as the Venus linker between receptor components and the BiFC fragment because this sequence had previously been used in BiFC experiments targeting the CGRP receptor [15]. For fusion constructs in which the BiFC fragments were attached to the N-termini of the receptors, we utilized the amino acid sequence “GSAGSAGSA” as the Venus linker because this has previously been used to tether the extracellular domains of CLR and RAMP1 [24]. To distinguish between the position of the mVenus fusion protein in naming the receptor constructs, we use a scheme where the position of the fusion is indicated by a prefix (either C or N, indicating C or N-terminal fusion, respectively) and by its position within the name of the construct. For instance, CLR-C-V_1–154_ refers to a construct in which V_1–154_ is fused to the C-terminus of CLR. Likewise, N-V_155–238_-RAMP1 refers to a construct in which V_155–238_ is fused to the N-terminus of RAMP1 (Figure 1).

### DNA modification

DNA was modified using an InFusion kit (Cat# 639650, Takara Biosciences, San Jose, CA, U.S.A.) according to manufacturer's specifications, with inserts and primers purchased from Integrated DNA Technologies (Coralville, IA, U.S.A). Briefly, linearized vectors were created from our WT receptors encoded in pcDNA3.1 through PCR reactions in which the primers were targeted to the desired insert site; all oligonucleotides used in this project are available in the supporting information. For C-terminal fusion of V_1–154_ to CLR, this linearization step also incorporated DNA overhangs on either side of the insert site; overhangs were complementary to the desired insert. The resultant product was treated with *Dpn*I for 5 min at 37°C to remove template DNA from the product. This product was run on a 1% agarose gel in tris borate EDTA with SYBR Safe (1:10,000; Cat# S33102, ThermoFisher, Waltham, MA, USA) to confirm amplification and linearization. Gels were visualized in a GelDoc (BioRad, Hercules, CA, U.S.A.). If a single band was detected, the PCR product was incubated with HiFi PCR CloneAmp (Cat# 639298, Takara Biosciences) at 37°C for 30 min followed by an incubation at 80°C for 20 min. The product was then diluted to between 50 and 100 ng/µL in UltraPure DNase/RNase-free H_2_O (Cat# 1523-102, Life Technologies, New Zealand). If multiple bands were detected, then the band of the desired molecular weight was excised from the gel, and the DNA extracted from the gel using a NucleoSpin Gel and PCR Clean-up kit according to manufacturer's instruction (Cat# 740609 Machery-Nagel, Allentown, PA, U.S.A.), followed by a dilution to between 50 and 100 ng/µL as above.

PCR was also performed to amplify insert DNA. With the exception of C-terminal fusion of V_1–154_ to CLR, complementary overhang sequences were also introduced in this PCR step. The resultant product was electrophoresed as above to confirm amplification, and the product either treated with PCR CloneAMP, or excised and extracted from the gel as above. These products were diluted to between 20 and 30 ng/µL in UltraPure H_2_O. All oligonucleotides used in these reactions are available in the supporting information.

The InFusion reaction was performed according to manufacturer's protocol, adding 50–100 ng insert DNA, 20–30 ng vector DNA, 1 µL of InFusion reaction mix, and UltraPure H_2_O, for a total volume of 5 µL. The mix was incubated at 50°C for 15 min, then placed on ice.

Ultracompetent *E. coli* (Cat# C2987I, New England BioLabs, Ipswich, MA, USA) were thawed on ice and 2 µL of resultant InFusion DNA added to 40 µL of cells. Cells were gently mixed then incubated on ice for 30 min, followed by a 45 s incubation at 42°C. Cells were returned to ice for 2 min, after which 360 µL of SOC broth (Takara BioSciences) was added to the cells, and the cells incubated for 1 h at 37°C with occasional mixing. After incubation, 100 µL of the broth was spread on an agar plate containing 100 µg/mL of ampicillin, which was then left to incubate overnight at 37°C in room air to allow colony formation.

Colony PCR using T7 and BGH primers followed by gel electrophoresis was performed to investigate whether colonies contained the insert of interest. The resulting DNA was sequence verified by Sanger sequencing (performed by the DNA sequencing facility at the Centre for Genomics, Proteomics, and Metabolomics at the University of Auckland School of Biological Sciences). Colonies containing the desired DNA sequence were then amplified, then the DNA extracted using a commercial maxiprep kit as per kit protocol (Cat# 74041, Machery-Nagel).

### Cell culture and transfection

HEK293S and Cos7 cells were used. In our hands, these generally have little/no endogenous CLR, CTR, or RAMPs [21,25], although during the course of this project there was a low level of CTR expression detected in our HEK293S cells [26], hence performing the experiments in two separate cell backgrounds. Cells used were between passage 10 and 30 and cultured as previously described [21]. Briefly, cells were cultured in DMEM (Cat# 11995065, ThermoFisher) containing 8% heat inactivated New Zealand origin fetal bovine serum (Cat# 10091-148, Gibco, Waltham, MA, U.S.A) in a humidified 37°C incubator with 5% CO_2_/95% air. For cAMP experiments, Cos7 or HEK293S cells were seeded at 20,000 cells per well in 96-well Spectraplates (Cat# 6005658, Revvity, Waltham, MA, U.S.A.) or CellBind plates (Cat# COR3300, Corning, Corning, NY, U.S.A.), respectively; in both cases, cells were transfected 24 h after seeding [17]. For immunofluorescence experiments, HEK293S cells were seeded at 12,500 cells per well in 96-well black PhenoPlates (Cat# 6055300, Revvity) which were coated in-house with poly-D-lysine (Cat# FAL354210, In Vitro Technologies), and transfected 36 h after seeding [17]. Cos7 cells were plated at 15,000 cells per well in 96-well black PhenoPlates and transfected 24 h after seeding [27]. All cells were transfected using polyethyleneimine with a 1:1 plasmid mass ratio of CTR/CLR to RAMP1 (leading to molar ratio of 1:1.5 for CTR/CLR:RAMP1, respectively). When investigating the ability of CTR to signal or translocate to the cell surface in the absence of RAMPs, cells were transfected with CTR and vector control at a plasmid mass ratio of 1:1. Where negative controls were required for imaging experiments, wells were transfected with empty vector at an equivalent total DNA per well as for receptor transfected wells. For cAMP experiments, cells were left for 40–48 h following transfection before use. For immunofluorescence experiments, HEK293S cells were left for 24-32 h post-transfection, and Cos7 cells left for ∼40–48 h post-transfection before being used in experiments [17,27]. Different growth periods were used to obtain cell confluence that was optimal for the experiment in question.

### Experimental design

All experiments were independently replicated at least three times with duplicate, triplicate, or quadruplicate technical replicates. Each independent experiment (or *n*) is a biological replicate consisting of cells from distinctly passaged flasks, transfected using distinct transfection mixes, and either stimulated with distinct peptide dilutions or probed using distinct antibody dilutions. Technical replicates from each independent experiment were averaged prior to combining datasets. In cAMP experiments, a WT control was included on each plate, while in immunofluorescence experiments both a WT control and a vector control were included in all experiments. Transfections were randomly assigned to positions on the plate to remove potential bias from plate position effects.

### cAMP assays

Cells were stimulated with αCGRP with duplicate or triplicate technical replicates. Transfected cells were stimulated for 15 min, after which stimulation media was aspirated and replaced with ice-cold absolute ethanol. Ethanol was then evaporated and cAMP detected using the LANCE cAMP assay kit (HEK293S cells; PerkinElmer, no longer supplied) or a CisBio G_s_ dynamic kit (Cos7 cells; Cat#62AM4PEC, Revvity) as previously described, with the kit incubation period extended to 4 h [26,28]. These two kits give comparable results [26]. Plates were read using an EnVision microplate reader (Revvity) or ClarioStar (BMG LabTech, Ortenberg, Germany).

### Data analysis for cAMP assays

Data analysis was performed in GraphPad Prism 9 or 10 (GraphPad Software, San Diego, CA, USA; HEK293S or Cos7 data, respectively). cAMP values were interpolated from a standard curve that was included in each experiment. Concentration-response curves were fit using a 3-parameter logistic equation, from which pEC_50_ and E_max_ values were derived. In cases where agonism was weak, in some individual experiments agonism was quantifiable, while in others the agonism was unquantifiable. This is likely a consequence of our transient transfection system which produces day-to-day variability in receptor expression. A response was deemed quantifiable when at least two data points were above the response to media control; if this threshold was not met, we refer to the response as unquantifiable, and we did not attempt to fit a curve to the data. In figures and tables, we present data that reflect the majority of experiments i.e. if the majority of experiments showed an unquantifiable response, we present data without a curve fit. To avoid biasing data reporting, we describe data from the minority experiments in the associated table legends [29].

Data sets comprising pEC_50_ values were analyzed by repeated measures analysis of variance (ANOVA) tests (groups of four conditions with identical *n* numbers) or a mixed-effects model (groups of four conditions with differing *n* numbers) both with post-hoc Holm–Šidák analysis comparing the control value to each other value, or a paired Student's *t*-test (two conditions with identical *n* numbers); statistical testing was chosen based on the number of groups being compared. A Holm–Šidák analysis was chosen for post-hoc analyses because this analysis technique has high power to detect differences in cases where group sizes are relatively small. Raw E_max_ values were log-transformed and analyzed identically with the pEC_50_ values; this analytical approach considers the relative difference between E_max_ values within experiments rather than absolute difference [30,31]. In all cases, statistical significance was accepted when *P* < 0.05.

Due to inherent variation from transient transfection, data were normalized to the WT control in each experiment by setting 100% and 0% of WT response as the E_max_ and E_min_ of the WT curve in each experiment. Normalized curves were generated for presentation by combining the mean of data points from normalized individual experiments. Analyses were not performed on normalized data.

### Fixed cell fluorescence assays

Cell surface expression of constructs was detected using immunofluorescence protocols as previously described [28]; the intrinsic fluorescence of mVenus was directly detected in the same assay. Briefly, on the day of the experiment, transfection media was aspirated and replaced with 4% paraformaldehyde (PFA) in phosphate-buffered saline (PBS) and left to incubate for 20 min at room temperature with shaking, after which the cells were washed twice with PBS. Goat serum (10% in PBS; Cat# 16210064, Life Technologies) was used as a blocking agent; cells were blocked for 1 h at room temperature with shaking. Wells were aspirated, and cells incubated for 30 min at 37°C with mouse anti-myc diluted 1:250 (Cat# H-9658 [9E10], Lot D00123355, RRID AB_10682957, Merck, Kenilworth, NJ, U.S.A.) and rabbit anti-HA diluted 1:1000 (Cat# MA5-27915 [RM305], Lot WF3297242, RRID AB_2744968, ThermoFisher), both in PBS containing 1% goat serum. Following two wash steps, goat anti-mouse conjugated to AlexaFluor (AF)-647 diluted 1:1000 (Cat# A21235, RRID AB_2535804, Life Technologies), and goat anti-rabbit conjugated to AF-568 diluted 1:1000 (Cat# A11011, Lot 1778025, RRID AB_143157, Life Technologies) in 1% goat serum in PBS were added to all wells and the plate left to incubate for 1 h at room temperature. Wells were washed twice with PBS, then 100 µL of cell-based dye (either DAPI [Cat# D1306, ThermoFisher; final concentration of 300 nM in PBS] or Cell Mask Blue (CMB) [Cat# H32720, ThermoFisher; final dilution of 1:1000]) was added to the plate. Initial experiments used CMB as a cell marker however due to shipping constraints DAPI was used in later experiments. Cell-markers were not included in analyses and thus results from experiments using the different dyes were able to be compared. Plates were incubated at room temperature for 30 min. Wells were washed three times with PBS, then 100 µL of PBS was added to the wells before imaging in an Operetta high-content imaging device (Revvity) or an Opera Phenix high-content imaging device (Revvity). Images were acquired from at least three separate positions in each well; positions were kept consistent within experiments. When using an Operetta, images were acquired using 20x and 40x lenses (numerical apertures of 0.75 and 0.6, respectively), with excitation filters 380/40, 500/20, 570/20, and 630/20 nm, and emission filters 445/70, 540/40, 615/50, and 705/110 nm used for imaging DAPI/CMB, mVenus, AF-568, and AF-647, respectively. When using an Opera Phenix, images were acquired using a 20x lens (numerical aperture of 0.4) with excitation lasers at 375, 488, 561, and 637 nm, and emission filters of 475/80, 525/50, 600/60, 705/110 nm, for imaging DAPI, mVenus, AF-568, and AF-647, respectively. The antibodies and filters used were chosen to minimize spectral overlap. During imaging, the fluorescence signal was kept within the limits of the detector to avoid saturating individual pixels. The results from the Opera Phenix and the Operetta were comparable thus analyses were combined [17,28].

### Data analysis for fixed cell fluorescence assays

Data analysis was performed using Signals ImageArtist software (Revvity) using images captured at 20x magnification. Analysis was based on the integrated intensity approach of Grimsey, Narayan [32]. Data analysis was automated, meaning that the analysis pipeline was essentially blinded.

For the detection of the myc-tag (myc-RAMP1) or the HA-tag (HA-CLR or HA-CTR), a common threshold building block of 0.5 arbitrary units was applied to the AF-647 (myc) or AF568 (HA) channels with a minimum area of 200 or 400 µm for HEK293S or Cos7 cells, respectively. This difference in minimum size is due to each individual Cos7 cell being larger than an individual HEK293S cell. This combination of thresholding enabled selection of regions in the images with fluorescent signal above background levels, and reduced interference from small bright spots arising from cell debris. This AF-647 thresholding acted to select regions within the image that displayed myc-like immunoreactivity above background levels, which we use as a proxy for RAMP1 detection in transfected cells. RAMP1 does not traffic to the cell surface alone, therefore this signal represents expression of a complete receptor complex. We calculated the mean intensity of both mVenus and AF-647 in these regions. This AF-568 thresholding acted to select regions within the image that displayed HA-like immunoreactivity above background levels, which we use as a proxy for CLR or CTR detection in transfected cells. The CGRP receptor is an obligate dimer, so HA-detection is likely to represent the full receptor complex. In contrast, cells transfected with the AMY_1_ receptor that are positive for HA-like immunoreactivity probably represent a combination of the AMY_1_ receptor complex and free CTR expression [11,26,33]. In these regions, we derived the mean intensity of AF-568. We reasoned that not all cells identified through this method would contain both receptor components, and thus we did not derive the intensity of mVenus.

For each technical replicate, the mean intensity value from each field of view was averaged across all other values from that well, creating one mean intensity value for each technical replicate. The technical replicates within an experiment were then averaged to create a mean value for the biological replicate. These mean intensity values from biological replicates were then combined for analysis.

For AF-647 and AF-568 analysis, the combined, log-transformed fluorescence intensity values were analyzed by repeated-measures one-way ANOVA with post-hoc Dunnett's test, comparing each condition to the WT receptor condition. This comparison was performed because we were interested in whether fusion of the split mVenus protein altered the cell-surface expression of the receptor-complex relative to the WT receptor. Due to variability associated with transient transfection, log transformation was used to enable comparisons between relative fluorescence differences rather than absolute fluorescence levels. Background corrected fluorescence quantification values were derived for presentation by subtracting the mean value from vector control wells from each other condition within each experiment.

For mVenus analysis, we explored whether the mVenus fluorescence arising from the interaction between V_1–154_ and V_155–238_ was above background levels. The combined, log-transformed mVenus values were analyzed by one-way repeated-measures ANOVA with post-hoc Dunnett's analysis, comparing values from WT transfected cells to each other condition. Background-corrected values were derived for presentation by subtracting the mean mVenus signal in the WT receptor wells from each other condition within the experiment. We also compared the mVenus fluorescence from C-terminal and N-terminal mVenus BiFC fusions within receptors and cell-types. Background corrected, log-transformed mVenus fluorescence intensity values were compared using unpaired Student's *t*-tests. In all cases, statistical significance was accepted when *P* < 0.05.

### Assays comparing mVenus fluorescence in live and fixed HEK293S cells

Assays were performed 24 h after transfection. Wells were aspirated and cells washed once with warm PBS. Cells were then incubated for 30 min at 37°C in 50 µL of imaging media (phenol-red free DMEM [ThermoFisher, Cat# 21063029] + 0.1% BSA). The mVenus fluorescence was then imaged in a Phenix high-content imager (pre-warmed to 37°C) using a 20x objective lens. Wells were then aspirated and 4% PFA added for 20 min at room temperature. Wells were washed twice with PBS, after the second wash 200 µL of PBS was added to the wells and the plate returned to the Phenix for reimaging. The intensity of mVenus in each image was then calculated in Signals ImageArtist using a common threshold building block of 0.5 arbitrary units followed by a calculation of pixel intensity. Data were compared by two-way repeated measures ANOVA with a Fisher's LSD test, comparing fixed and live cells within and between fusion orientations.

### Internalization assays in fixed HEK293S cell time-course format

This experimental protocol used a previously characterized Cy5-labelled human αCGRP (Cy5 label on residue 3), which has similar pharmacology to the unmodified peptide [17]. Assays were performed 24 h after transfection. Wells were aspirated and cells washed once with warm PBS. Cells were serum starved in 50 µL of imaging media for 30 min then exposed to 10 nM of Cy5-αCGRP for the defined length of time, after which they were aspirated, washed twice with 50 µL of imaging media, and fixed with 4% PFA for 20 min at room temperature. Cells were then probed using anti-HA antibodies as described in the “fixed cell fluorescence assay” section with the exception that PBS was replaced with TBS containing 0.1% Tween in all instances to allow detection of intracellular (internalized) receptors. Cells were imaged in a Phenix high-content imager, using a 63x lens in confocal mode. Twenty fields of view were captured per well. Internalization was quantified by detecting the formation of puncta in the Cy5 channel as described previously [17], with the modification that the initial step of finding cells used the AF-568 channel (HA-tag).

## Results

### Effect of BiFC fusion proteins on cAMP production

We first tested functionality of receptor constructs in cAMP signaling assays; cAMP was chosen because it is the canonical pathway through which these receptors signal [11]. In these experiments, we tested the ability of αCGRP to stimulate cAMP production at WT receptors, and compared that with receptors which incorporated single BiFC fusion constructs (for example CLR-C-V_1–154_/RAMP1) and receptors which incorporated both BiFC fusion constructs (for example CTR-C-V_1–154_/RAMP1-C-V_155–238_; Figure 1). CLR does not traffic to the cell surface or act as a receptor in the absence of a RAMP; however, CTR is a functional receptor in the absence of RAMP co-expression. Therefore, we also characterized the effect of V_1–154_ fusion on CTR alone. Experiments were performed in two different cell types to investigate the influence of cell background on results.

**Figure 2:**
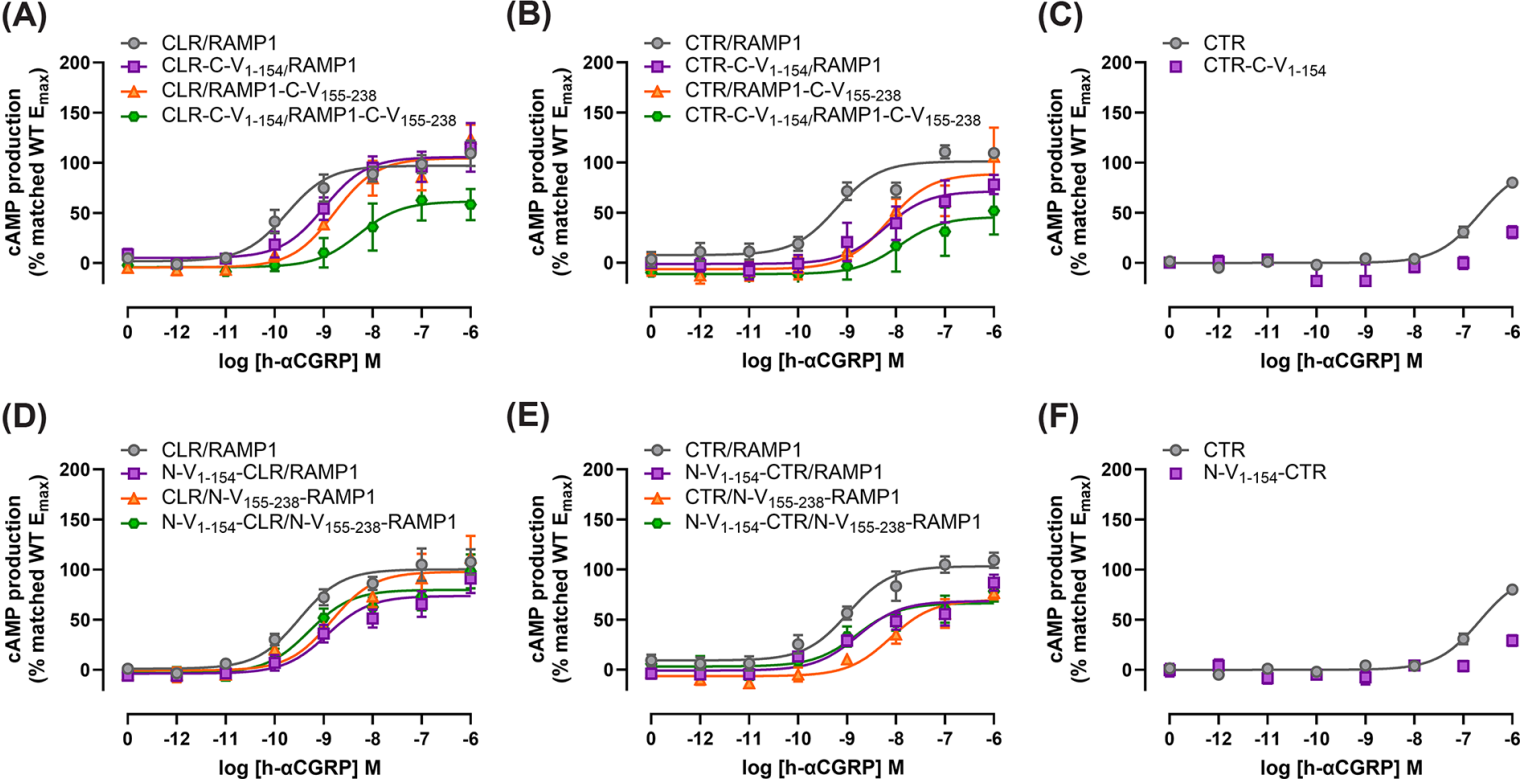
Concentration-response curves for h-αCGRP-stimulated cAMP production at CGRP and AMY_1_ receptors in Cos7 cells Results from CGRP or AMY_1_ receptors incorporating C-terminal (**A–C**), or N-terminal (**D–F**) mVenus BiFC fusion constructs in transiently transfected Cos7 cells (15-min stimulation duration). Each point is the mean ± s.e.m. of at least three independent experiments performed in duplicate or triplicate, for exact *n* numbers see Table 1. There is one additional experiment at CTR-C-V_1–154_ and N-V_1–154_-CTR in which αCGRP elicited a measurable curve, this has been excluded from the presented data set and is instead described in the legend of Table 1. Panels C and F present the same control CTR curve, however for ease of comparison we have presented this data in two panels.

Single C-terminal fusions had a small impact on the potency of αCGRP at the CGRP and AMY_1_ receptors (<10-fold difference from WT; Figure 2 and Supplementary Figure S1–3). In Cos7 cells for both CTR-C-V_1–154_/RAMP1 and CTR/RAMP1-C-V_155–238_, these differences were statistically significant (Table 1, Supplementary Figure S1). There were very few differences in the E_max_ of these receptors; however, in HEK293S cells, CTR/RAMP1-C-V_155–238_ had a statistically significant lower E_max_ than the WT AMY_1_ receptor (Supplementary Table S1 and Figure S3).

**Table 1:** pEC_50_ and E_max_ values for h-αCGRP-stimulated cAMP production at fusion constructs encoding V_1–154_ and/or V_155–238_ attached to either the N or C terminus of CGRP or AMY_1_ receptor subunits in transiently transfected Cos7 cells

Fusion position	Receptor	WT receptor			V_1–154_ fusion alone			V_155–238_ fusion alone			V_1–154_ and V_155–238_ fusions		
		pEC_50_	E_max_	*n*	pEC_50_	E_max_	*n*	pEC_50_	E_max_	*n*	pEC_50_	E_max_	*n*
C-terminal	CLR/RAMP1	9.69 ± 0.32	100	5	9.09 ± 0.25	100.30 ± 11.52	5	9.00 ± 0.19	98.10 ± 8.69	5	8.08 ± 0.29*	74.95 ± 10.24	5
	CTR/RAMP1	9.31 ± 0.21	100	6	8.41 ± 0.33*	81.16 ± 9.65	6	8.62 ± 0.28*	83.49 ± 7.63	6	7.52 ± 0.27*	71.47 ± 15.29	6
	CTR	6.63 ± 0.07	100	4	NC	NC	3^#^	NA	NA	NA	NA	NA	NA
N-terminal	CLR/RAMP1	9.58 ± 0.12	100	5	9.20 ± 0.22	78.26 ± 7.52	5	9.31 ± 0.23	91.79 ± 12.2	5	9.25 ± 0.13	84.89 ± 6.74	5
	CTR/RAMP1	9.10 ± 0.12	100	6	8.92 ± 0.36	74.02 ± 5.16*	6	8.16 ± 0.20*	74.86 ± 6.96	6	8.83 ± 0.08	77.34 ± 8.01	6
	CTR	6.63 ± 0.07	100	4	NC	NC	3^#^	NA	NA	NA	NA	NA	NA

Results are the mean ± s.e.m. of *n* independent experiments performed in duplicate or triplicate. E_max_ values presented as a percentage of the matched WT curve included in each experiment. Values were compared across rows. pEC_50_ values were analyzed by repeated measures ANOVA (groups of four conditions with identical *n* numbers) or a mixed-effects model (groups of four conditions with differing *n* numbers) with post-hoc Holm–Šidák tests, comparing the WT value to each other value. Non-normalized E_max_ values were log-transformed to enable paired testing, before being analyzed identically with the pEC_50_ values. Statistical significance was accepted when *P* < 0.05, and is indicated by an * (asterisk). NA – not applicable because we were testing the effect of fusing V_1–154_ to CTR in the absence of RAMP1. NC – no curve could be fit to the data. ^#^ There was one independent experiment where αCGRP was able to stimulate detectable cAMP production at these constructs, with a pEC_50_ of 6.37 at CTR-C-V_1–154_, and 6.74 at N-V_1–154_-CTR, and an E_max_ of 75.43% and 54.83% of WT at CTR-C-V_1–154_ or N-V_1–154_-CTR, respectively.

In both Cos7 and HEK293S cells, co-expression of C-terminal BiFC-fusion proteins (being CLR-C-V_1–154_/RAMP1-C-V_155–238_ or CTR-C-V_1–154_/RAMP1-C-V_155–238_) resulted in a statistically significant ∼10-to-70-fold reduction in the potency of αCGRP (Figure 2 and Supplementary Figure S1–3, Table 1, and Supplementary Figure S1). In HEK293S cells, these receptors also had a statistically significant reduction in E_max_ compared with the relative WT receptors (Supplementary Table S1), while in Cos7 cells the E_max_ trended to being lower that WT receptors, but this did not reach statistical significance (Table 1).

Considering the N-terminal fusions, there was a small (<10-fold) but statistically significant decrease in the potency of αCGRP at CTR/N-V_155–238_-RAMP1 in Cos7 cells, and a small but statistically significant reduction in the E_max_ of αCGRP at N-V_1–154_-CLR/RAMP1 in HEK293S, and at N-V_1–154_-CTR/RAMP1 in Cos7 (Supplementary Figure S1–3). Other combinations of single N-terminal fusion constructs did not significantly affect the signaling of αCGRP at either the CGRP or AMY_1_ receptors (Figure 2 and Supplementary Figure S1–3, Table 1 and Supplementary Table S1). Interestingly, when considering the combined N-terminal fusion constructs (e.g. N-V_1–154_-CLR/N-V_155–238_-RAMP1), there was no significant impact on αCGRP signaling at either receptor in either cell-line (Figure 2 and Supplementary Figure S1–3, Table 1 and Supplementary Table S1).

Considering CTR alone, in HEK293S cells there was no significant difference between αCGRP signaling at WT CTR and CTR-C-V_1–154_, however αCGRP trended towards being less potent and having a lower E_max_ at N-V_1–154_-CTR relative to WT CTR; differences were not statistically significant (Supplementary Figure S2 and Table S1). In Cos7 cells, we were unable to fit a curve to data from αCGRP at CTR-C-V_1–154_ or N-V_1–154_-CTR; however, 1 µM αCGRP did elicit a robust response at these receptors indicating that higher concentrations may have allowed a full curve to be fit. We also tested calcitonin as an agonist at CTR alone, as it is more potent than αCGRP at this receptor. Calcitonin was less potent at CTR-C-V_1–154_ (∼3-fold less potent) and N-V_1–154_ (∼10-fold less potent) than at WT CTR; this difference, though small, was statistically significant (Supplementary Figure S4).

### Effect of BiFC fusion on cell surface expression

We next tested whether the BiFC fusion influenced the cell surface expression of receptor complexes. When expressed alone, RAMP1 is trapped intracellularly, but traffics to the cell surface upon co-expression with either CLR or CTR [11], thus we used detection of myc-RAMP1 as a measure of cell-surface expression of the full receptor complex. We also included an antibody against HA to detect the HA-tag on CLR and CTR to ensure the co-expression of RAMP1 with each GPCR; this analysis supplemented the analysis of myc detection. This antibody-based detection method only detects receptors on the cell surface, as opposed to receptor expression within the cell [17,28]. Generally speaking, most conjugations had limited effects on cell surface expression, and those that did have a statistically significant effect only caused modest changes to expression.

**Figure 3:**
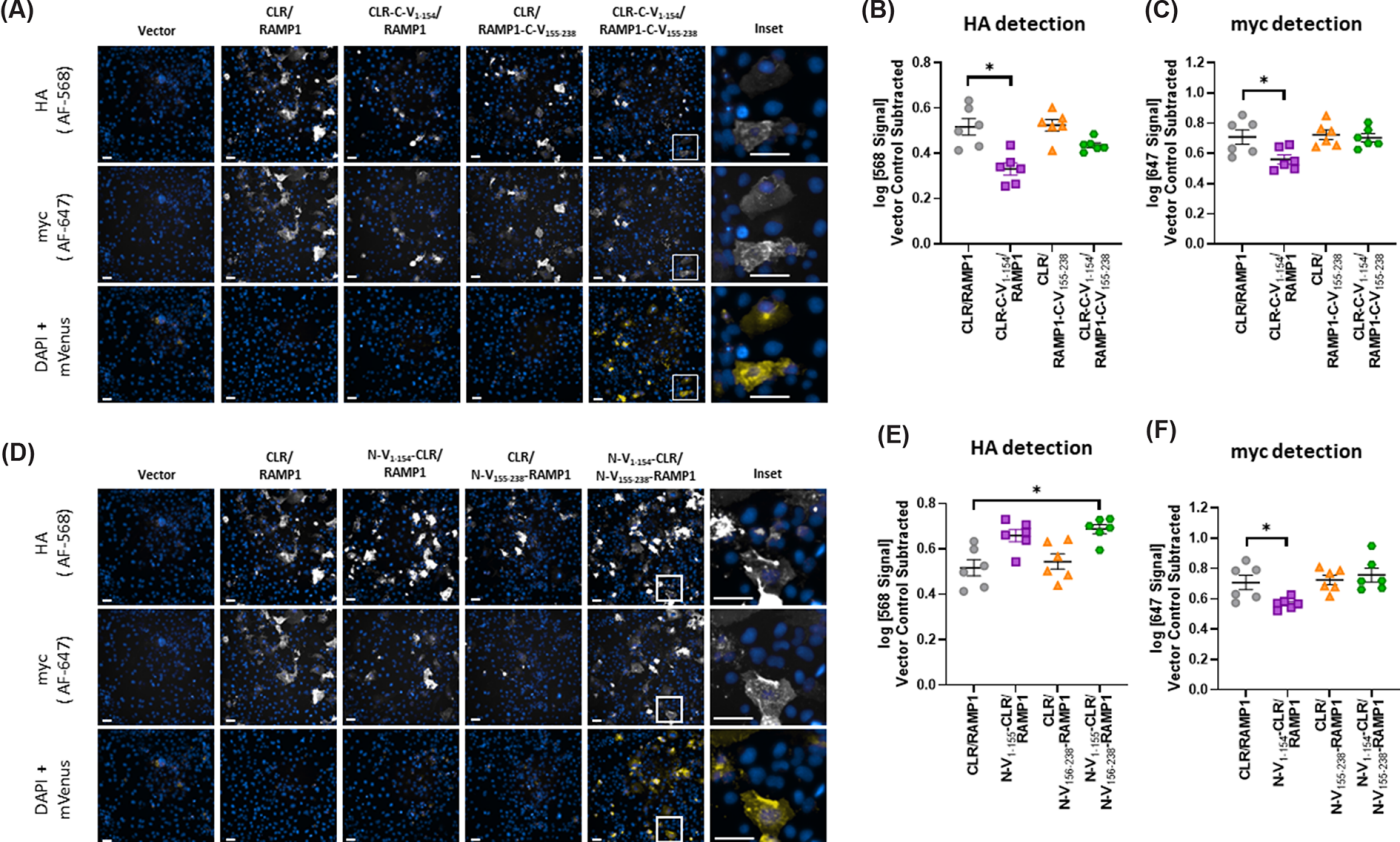
Cell surface expression and mVenus fluorescence of CLR complexes in Cos7 cells Detection and quantification of cell surface expression and mVenus fluorescence for CLR-based receptor complexes in transfected Cos7 cells. (**A–C**) are C-terminal fusion constructs, (**D–F**) are N-terminal fusion constructs. (**A,D**) Fluorescence results for antibody-based detection of AlexaFluor (AF)-568 and AF-647 (HA-tag and myc-tag, respectively), intrinsic mVenus fluorescence, and a cell marker (DAPI). AF channels are white, mVenus is yellow, and the cell marker (DAPI) is blue. Each column within a panel shows the same well and well position; all images within a panel are from the same independent experiment. Images are representative of six independent experiments performed in duplicate, triplicate, or quadruplicate. Each image is one field of view within a well, imaged at 20x. The brightness of images was modified to facilitate visualization on screen and in print; modifications were kept consistent within experiments and channels and care was taken during the acquisition process to make sure data did not saturate the detector. Scale bars indicate 50 µm. (**B,C,E,F**) quantified intensity of the HA (AF-568; B, E) or myc (AF-647; C, F) signal within each independent experiment. Each point is an independent experiment; lines indicate mean ± s.e.m. * indicates *P* < 0.05 compared with WT as determined by repeated measures one-way ANOVA with a post-hoc Dunnett's test comparing all conditions to WT.

We first describe the data for C-terminal fusion constructs for CLR-based receptors. All combinations were detected on the cell surface (Figure 3A, Supplementary Figure S5A). The CLR-C-V_1–154_/RAMP1 receptor showed significantly less HA and myc immunoreactivity than the WT CGRP receptor in both cell lines (Figure 3B,C, Supplementary Figure S5B,C), suggesting reduced cell surface expression. In HEK293S cells, CLR/RAMP1-C-V_155–238_ displayed slightly higher HA immunoreactivity than the WT CGRP receptor; there was a trend for a similar result when analyzing myc immunoreactivity; however, this did not reach statistical significance (Supplementary Figure S5,B,C). In Cos7 cells, these combinations were not different from WT (Figure 3B,C).

As with C-terminal fusions, all combinations of N-terminal fusion constructs were detected on the cell surface (Figure 3D, Supplementary Figure S5D). In both HEK293S and Cos7 cells, there was a significant decrease in myc immunoreactivity when comparing N-V_1–154_-CLR/RAMP1 to the WT CGRP receptor. In contrast, in Cos7 cells N-V_1–154_-CLR/RAMP1 and N-V_1–154_-CLR/N-V_155–238_-RAMP1 appeared to have increased HA immunoreactivity as compared with WT (Figure 3E,F). This suggests that the increased detection of HA and reduction in myc-detection with N-V_1–154_-CLR may be due to the extension of the N-terminus of the CLR construct, leading to increased epitope availability for HA-binding antibodies, and epitope masking of the myc epitope rather than actual loss of cell surface expression.

**Figure 4:**
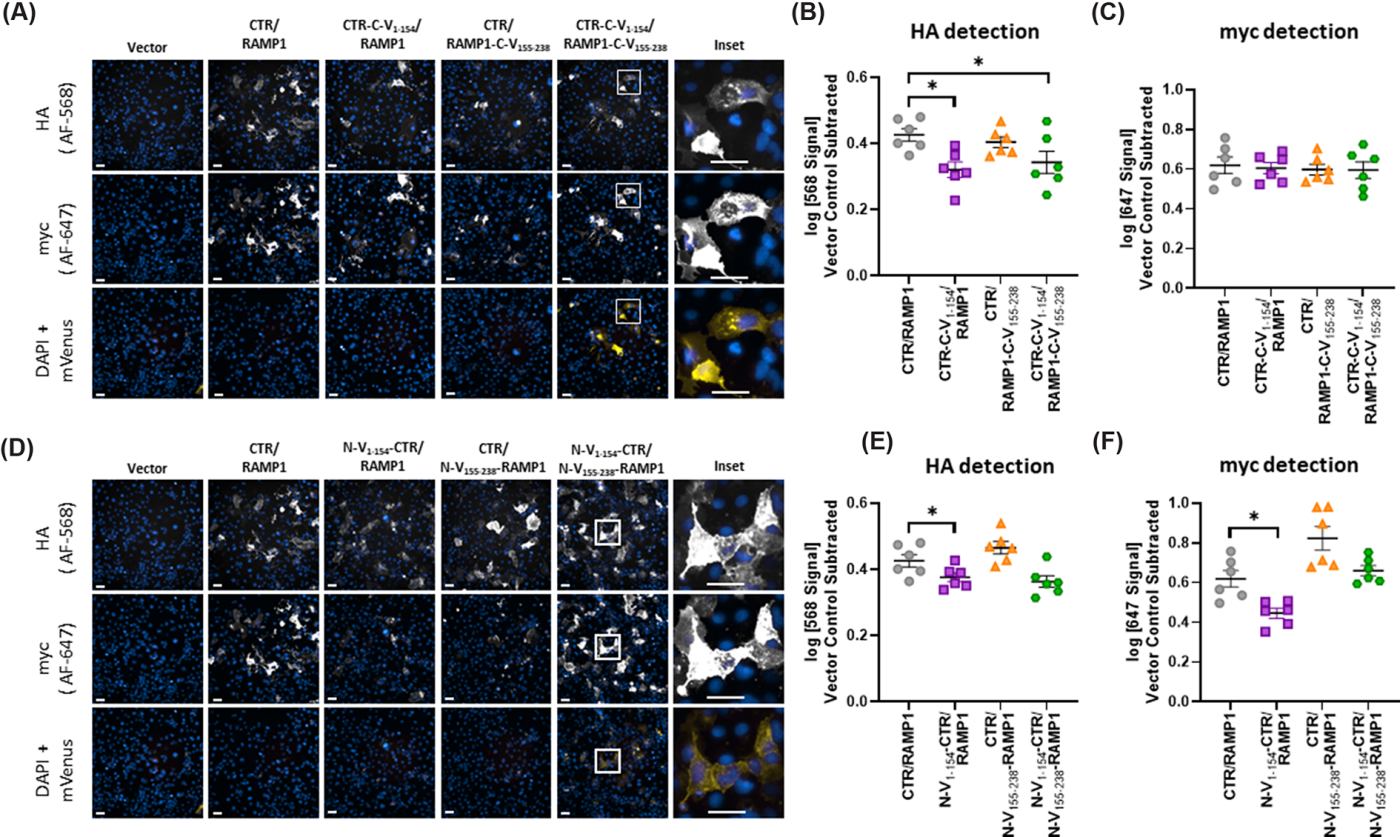
Cell surface expression and mVenus fluorescence of CTR complexes in Cos7 cells Detection and quantification of cell surface expression and mVenus fluorescence for CTR-based receptor complexes in transfected Cos7 cells. (**A-C**) are C-terminal fusion constructs, (**D-F**) are N-terminal fusion constructs. (**A,D**) Fluorescence results for antibody-based detection of AF-568 and AF-647 (HA-tag and myc-tag, respectively), intrinsic mVenus fluorescence, and a cell marker (DAPI). AF channels are white, mVenus is yellow, and the cell marker (DAPI) is blue. Each column within a panel shows the same well and well position, all images within a panel are from the same independent experiment. Images are representative of six independent experiments performed in duplicate, triplicate, or quadruplicate. Each image is one field of view within a well, imaged at 20x. The brightness of images was modified to facilitate visualization on screen and in print; modifications were kept consistent within experiments and channels and care was taken during the acquisition process to make sure data did not saturate the detector. Scale bars indicate 50 µm. (**B,C,E,F**) quantified intensity of the HA (AF-568; B, E) or myc (AF-647; C, F) signal within each independent experiment. Each point is an independent experiment; lines indicate mean ± s.e.m. * indicates *p* < 0.05 compared with WT as determined by repeated measures one-way ANOVA with a post-hoc Dunnett's test comparing all conditions to WT.

Equivalent experiments were performed using CTR-based receptors (Figure 4, Supplementary Figure S6). Again, all constructs were detected at the cell surface. Receptors comprising CTR-C-V_1–154_/RAMP1 displayed significantly lower HA immunoreactivity than the WT AMY_1_ receptor in HEK293S cells; there was also a trend for a reduction in myc immunoreactivity, but this was not statistically significant (Supplementary Figure S6B,C). In Cos7 cells, receptors comprising either CTR-C-V_1–154_/RAMP1 or CTR-C-V_1–154_/RAMP1-C-V_155–238_ had reduced HA immunoreactivity than the WT AMY_1_ receptor; however, there was no change to myc immunoreactivity (Figure 4B,C).

In HEK293S cells, both N-V_1–154_-CTR/RAMP1 and N-V_1–154_-CTR/N-V_155–238_-RAMP1 had a significant reduction in HA immunoreactivity compared with the WT AMY_1_ receptor; however, only N-V_1–154_-CTR/RAMP1 had significantly reduced myc immunoreactivity (Supplementary Figure S6E,F). Similar results were obtained in Cos7 cells, though N-V_1–154_-CTR/N-V_155–238_-RAMP1 was not significantly different from WT AMY_1_ with either myc or HA detection (Figure 4E,F). N-V_1–154_-CTR had an impact on the signaling of both αCGRP and calcitonin in both cell types, therefore we investigated its cell surface expression levels. Despite a trend for reduced expression in both cell types, the reduction was small and, in neither case, did this reach statistical significance (Supplementary Figure S7).

### Quantification of the fluorescent signal arising from BiFC mVenus

There was a clear signal in the mVenus channel when both GPCR and RAMP mVenus fusion constructs were transfected into cells. As this was not an antibody-based approach to detection, we did not differentiate between cell surface and intracellular fluorescence. The mVenus signal was evident for both C-terminal and N-terminal fusion constructs with both CLR and CTR based receptors. This signal was absent from cells transfected with vector control, WT constructs, or in cells transfected with only one of the BiFC fusion components (Figures 3 and 4, Supplementary Figures S5 and S6). This result was confirmed using one-way ANOVA with post-hoc Dunnett's comparing the quantified mVenus fluorescence intensity of WT receptor to all other transfection conditions within each receptor, fusion orientation, and cell type. In all cases, co-expression of V_1–154_ and V_155–288_ resulted in fluorescence that was consistent with mVenus protein expression and that was significantly above the signal detected in the WT background (Supplementary Figures S8 and S9).

**Figure 5:**
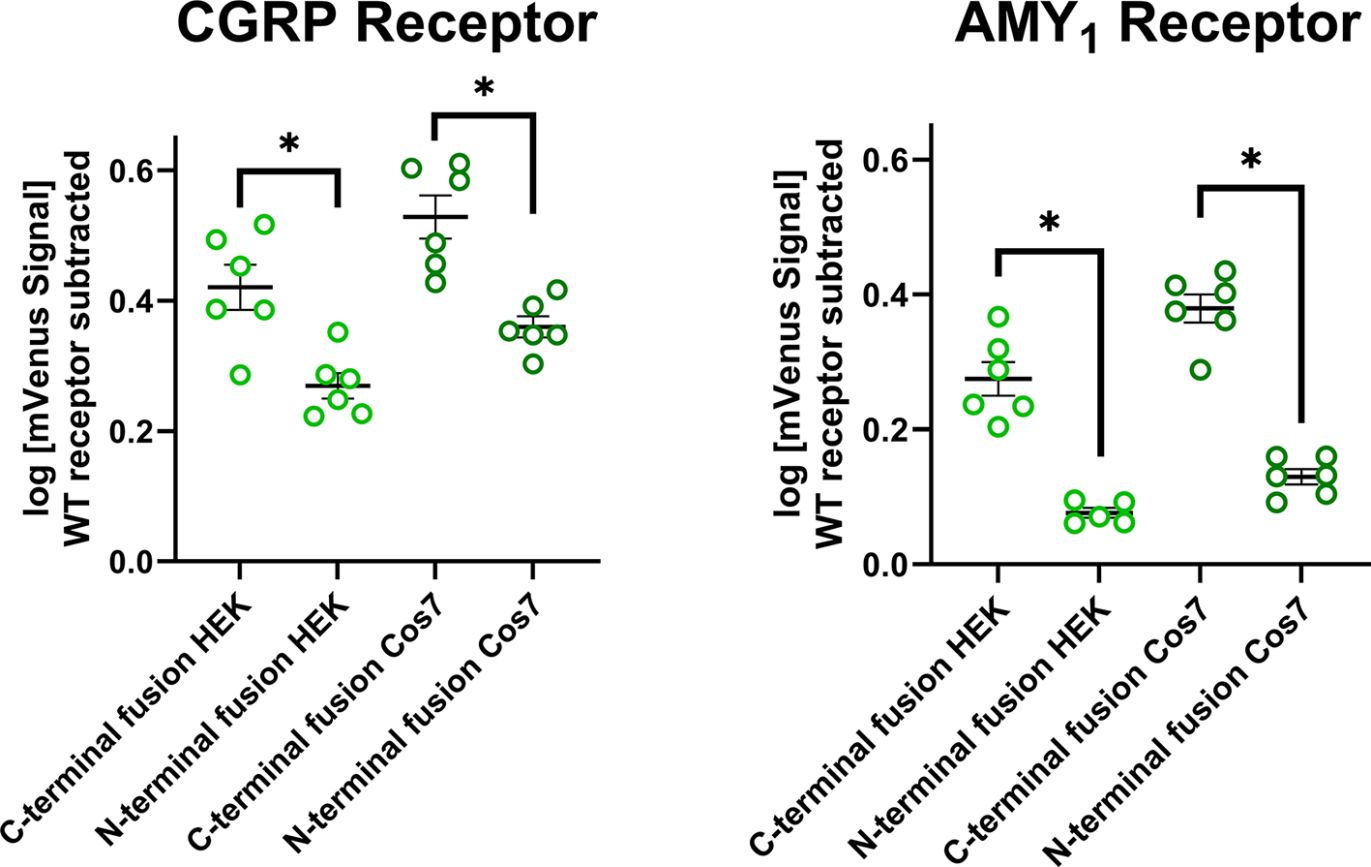
Quantification of mVenus fluorescence intensity for receptor fusions in HEK293S and Cos7 cells Quantification of mVenus fluoroescence intensity for each receptor and fusion orientation in HEK293S and Cos7 cells. Each data point represents an independent experiment, with lines denoting the mean ± s.e.m. The fluorescence from C-terminal fusion was compared with N-terminal fusion fluorescence within cell-types using Student's *t*-tests; * indicates *P* < 0.05.

We then compared mVenus signal intensity between the N-terminal and C-terminal fusion orientations for each receptor within cell-types. For both receptors and cell-types, C-terminal fusion resulted in significantly higher mVenus fluorescence than did N-terminal fusion as determined by Student's *t*-tests comparing orientation of fusion within receptor and cell-types (Figure 5).

**Figure 6:**
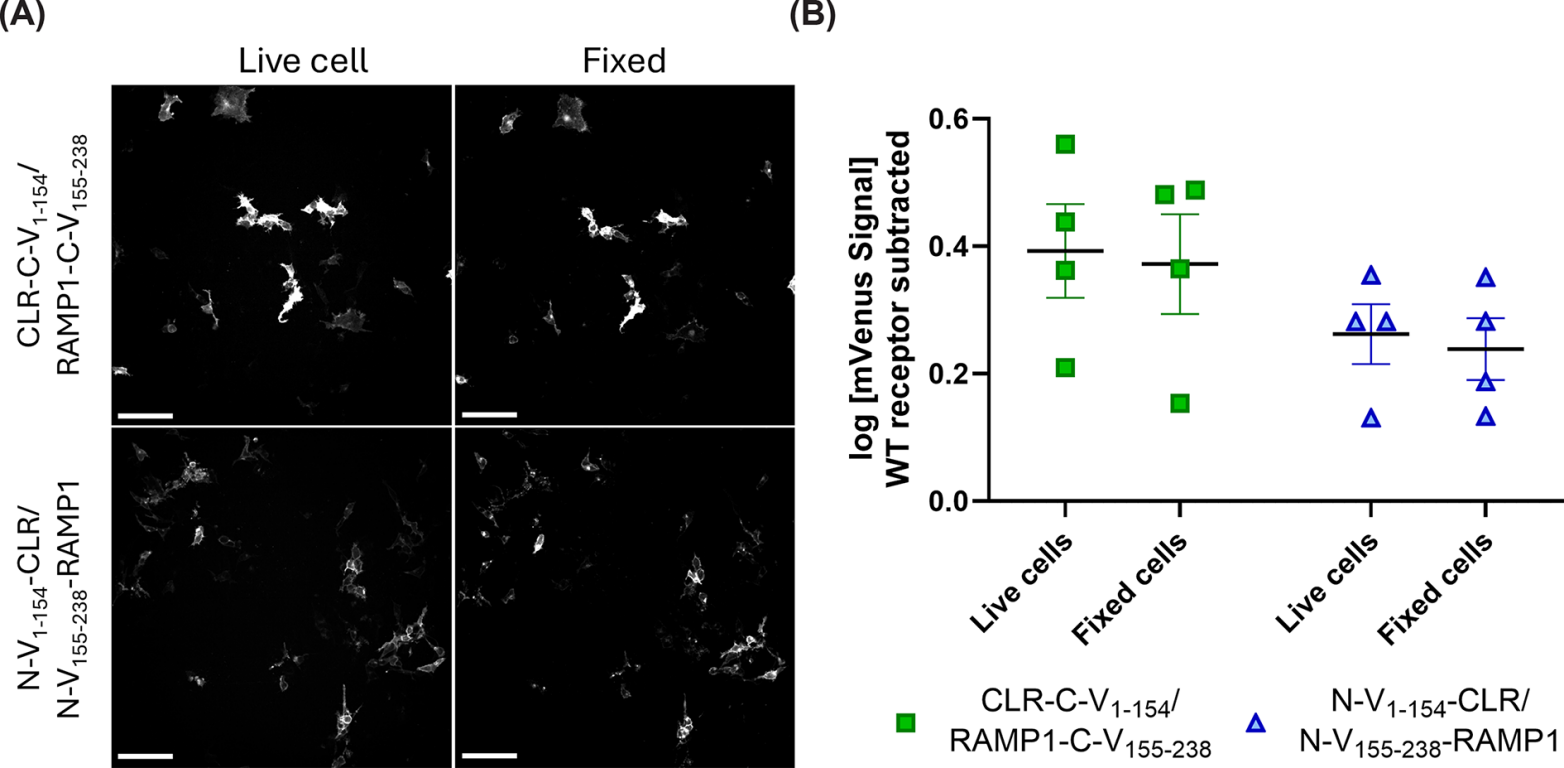
Detection and quantification of mVenus fluorescence in live and fixed HEK293S cells Detection and quantification of mVenus fluorescence in live and fixed HEK293S cells. (**A**) Images are the same well before and after fixation and are representative of four independent experiments with duplicate technical replicates. In the images, the BiFC signal is presented in greyscale. The brightness of images was modified to facilitate viewing on screen and in print; modifications were kept consistent across all images. During acquisition, care was taken not to saturate detectors. Scale bars represent 100 µm. (**B**) Quantification of mVenus intensity in live and fixed cells. Background-corrected values were derived for presentation by subtracting the mean mVenus signal in the WT receptor wells from each other condition within the experiment. Each data point represents an independent experiment with lines indicating mean ± s.e.m. Intensity was compared by two-way repeated measures ANOVA with Fisher's LSD test, comparing fixed and live cells within and between fusion orientations. There was no significant difference between live and fixed cells within fusion orientations; in all cases C-terminal fusions resulted in a significantly greater intensity than N-terminal fusions (comparison not shown on graph).

The N-terminal domains are extracellular, meaning that they are potentially more exposed to fixative and the subsequent pH changes/protein cross-linking that arises during the fixation procedure than the C-terminal fusions. We reasoned that this could interfere with the ability of reconstituted mVenus to fluoresce, as factors such as pH and PFA exposure are known to alter fluorescence output [34–36]. To explore this, we performed a limited subset of experiments focusing on the CGRP receptor (CLR/RAMP1), imaging cells before and after fixation with PFA. There was no significant difference in the mVenus signal when comparing between fixed and live cell conditions (Figure 6).

### Effect of BiFC on receptor regulation post-stimulation

**Figure 7:**
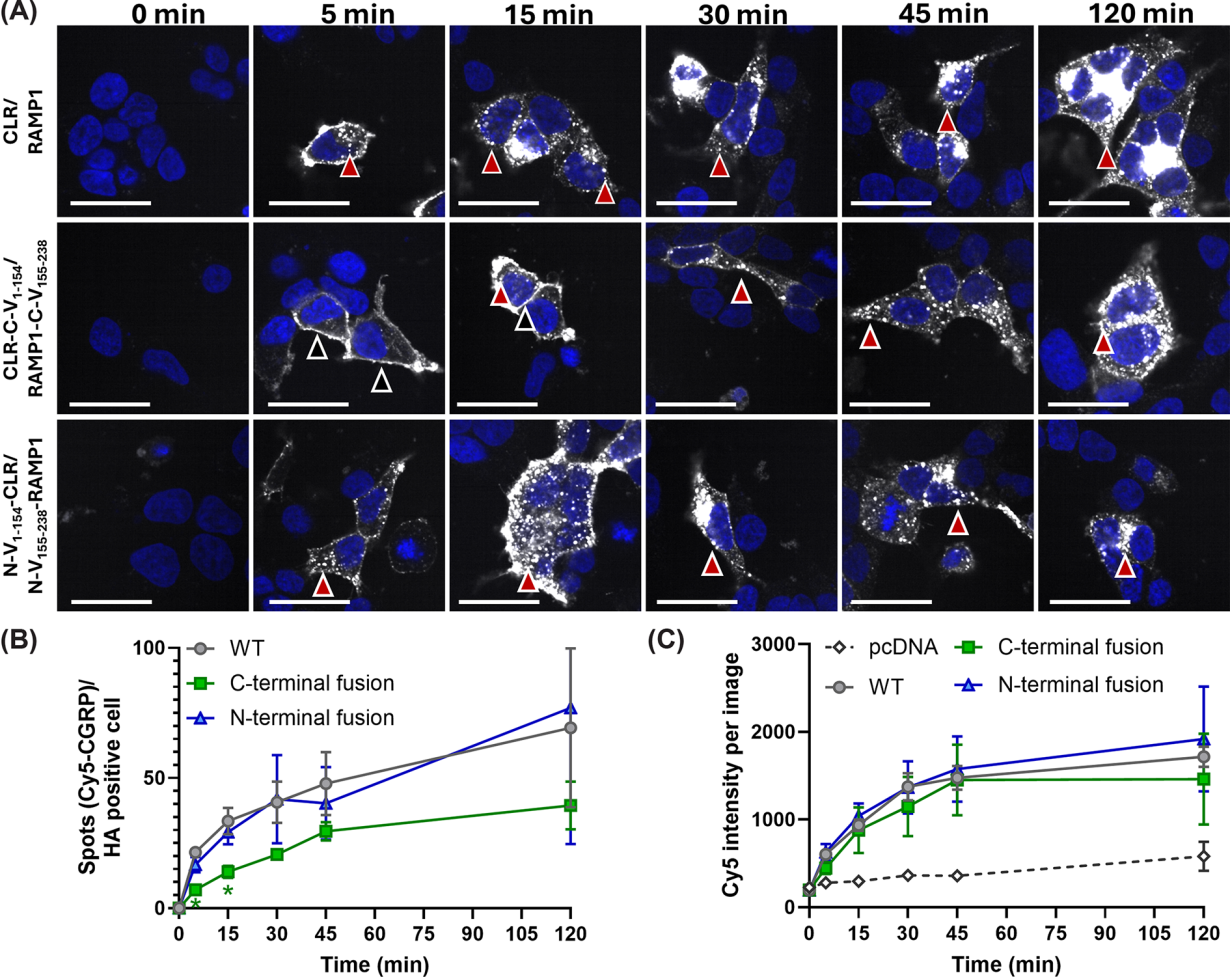
Localization and quantification of [Cy5]-αCGRP in HEK293S cells over time Localization and quantification of [Cy5]-αCGRP in HEK293S cells. (**A**) Localization of [Cy5]-αCGRP in HEK293S cells transfected with the WT CLR/RAMP1, C-terminal BiFC fusions, or N-terminal BiFC fusions across different time points. The Cy5 signal is white and DAPI is blue. Red-filled arrows indicate puncta formation, while black-filled arrows indicate binding to the cell surface. Scale bars indicate 100 µm. The brightness of images was modified to facilitate viewing on screen and in print; modifications were kept consistent across all images. During acquisition, care was taken not to saturate detectors. Scale bars represent 100 µm. (**B**) Quantification of puncta formation over time. Results from pcDNA-transfected wells are not included in this graph as results were normalized to the number of AF-568 positive cells in each image and as there were no cells expressing the HA-tag to detect, it was not possible to calculate a value. * indicates *P* < 0.05 compared with WT as determined by repeated measures one-way ANOVA with a post-hoc Dunnett's test comparing all conditions to WT at each time point. (**C**) Quantification of total Cy5 intensity as a proxy for ligand binding over time. There were no significant differences between receptor transfected conditions as determined by repeated measures one-way ANOVA with a post-hoc Dunnett's test comparing all conditions to WT.

Internalization of the CLR/RAMP1 complex following stimulation with CGRP is well documented, hence we explored whether BiFC fusions influenced the ability of the receptor complex to internalize [17,37,38]. The CTR/RAMP1 complex does not internalize in response to ligand stimulation, hence only CLR/RAMP1 was investigated [17,18]. To detect receptor internalization, we used a Cy5-labelled CGRP peptide which has similar pharmacology to the native CGRP. Using this peptide, we were able to quantify the formation of puncta within cells as a measure of receptor internalization [17]. The WT, C-terminal fusion constructs, and N-terminal fusion constructs all showed puncta formation in response to stimulation with Cy5 CGRP; however, C-terminal fusion constructs had a slower onset of puncta formation (being significantly less than WT at 5- and 15-min post peptide addition), while N-terminal fusion constructs did not show a significant difference when compared with WT (Figure 7A,B). This did not appear to be due to major differences in receptor number or peptide binding, as the total Cy5 intensity in each image (used here as a proxy of receptor-bound ligand) did not differ between constructs (Figure 7C). As such, it appears that C-terminal fusion does not interfere with binding, but can interfere with receptor internalization. In contrast, N-terminal fusion doses not interfere with internalization or binding.

## Discussion

CGRP-responsive receptors comprise either CLR or CTR in complex with RAMP1. As well characterized heterodimers, these receptors were chosen as models to investigate the positional effect of fluorescent protein fusion in BiFC experiments. In the present study, we show that N-terminal BiFC fusion is less disruptive to cell signaling than C-terminal fusion, and is still capable of producing fluorescent signal.

Generally, data between the HEK293S and Cos7 cell backgrounds were comparable, which is similar to previous literature [26,29]. CGRP (CLR/RAMP1) and AMY_1_ (CTR/RAMP1) receptors which incorporated the full complement of C-terminal BiFC fusion showed a reduction in potency and E_max_ in cAMP signaling (Figure 2, Supplementary Figure S1–3, Table 1). Both these receptors were detected on the cell surface at similar levels to WT receptor complexes (Figures 3 and 4, Supplementary Figures S5 and S4). Even when statistical analysis gave a significant difference, the changes in cell surface expression were modest and not likely to affect cell signaling [39]. The impaired signaling, combined with the retention of cell surface expression, suggests that the refolded mVenus on the intracellular side of these receptors interferes with the ability of intracellular effectors, such as G_α_s, to interact with the receptor, thereby reducing the activation of downstream signaling pathways. This is perhaps not surprising, given that the C-termini of GPCRs are known to be important for G protein interaction [40]. Introducing large proteins, such as fluorescent protein fragments, may sterically hinder these otherwise flexible intracellular regions and thereby interfere with G protein-coupling. Although C-terminal BiFC constructs have been created previously for the CGRP receptor, there was no investigation into the cell surface expression or cAMP signaling of these constructs [15].

Interference with coupling of intracellular proteins to receptors could also alter the regulation of these receptors by preventing the interaction between β-arrestin and the receptor. Although C-terminal BiFC CGRP receptors can recruit luciferase-fused β-arrestin in response to CGRP stimulation [15], there has been no comparison between WT and BiFC fusion constructs, and thus we do not know whether the fusion of the fluorescent protein to CLR alters the relative amount of arrestin recruitment. Receptor internalization and subsequent endosomal signaling are gaining prominence for their role in GPCR physiology [17,38], therefore new molecular tools should seek to avoid disrupting regulatory pathways. The C-terminal fusion orientation reduced internalization rates in addition to having a deleterious effect on cAMP production, therefore it is likely that the refolded mVenus interferes with the recruitment of intracellular effector molecules to the C-terminus of the receptor.

In contrast to C-terminal fusions, N-terminal fusion of BiFC components had less deleterious effects on cAMP signaling and internalization. Incorporation of both V_1–154_ and V_155–238_ did not alter the potency of αCGRP at either receptor; however, there was a trend towards a small reduction in the E_max_ of αCGRP at the AMY_1_ receptor in Cos7 cells. Single component N-terminal fusions were often well tolerated but in some cases caused effects; αCGRP had a statistically significant reduction in potency at the CTR/N-V_155–238_-RAMP1 (Cos7 cells), a significant decrease in E_max_ with N-V_1–154_-CLR/RAMP1 (HEK293S cells) and N-V_1–154_-CTR/RAMP1 (Cos7 cells) and both αCGRP and calcitonin had reduced signaling at the N-V_155–238_-CTR (αCGRP both cell types, calcitonin only tested in Cos7). This was unlikely to be due to altered cell surface expression, as changes arising from BiFC fusion were generally modest (Figures 3 and 4, Supplementary Figures S5 and S6) and in our systems minor changes to cell-surface expression generally do not affect cell signaling [39]. This reduction in E_max_ could have physiological relevance, as both CLR and CTR can interact with RAMP2 and RAMP3, and CTR can signal alone. Thus, if the end goal is to use this N-terminal fusion methodology in a more physiologically relevant system, such as genetically modified animals, it is important to consider that the interaction between V_1–154_-tagged receptors and other WT proteins may differ from interactions that occur with the native receptors and proteins. This could be particularly important for receptors like CLR, which is implicated in vascular and lymphatic development [41].

A limitation of our cAMP signaling work is that results were generated using end-point assays at a single time point (15 min stimulation). Future work could investigate whether BiFC fusion alters the kinetics of receptor activation, as there may be subtle differences in receptor activation that have been missed in our assays [42–44]. A further limitation is that all our signaling work was based on populations of cells, rather than individual cells. Future work could use genetically encoded sensors and microscopy to investigate signaling in single cells, for example, using FRET-based methods for measuring cAMP production [45–47]. The advantage of this approach is that it could restrict analysis to only cells expressing the full BiFC construct; however, rigorous controls would be needed to ensure that the reconstituted mVenus did not interfere with the FRET signal.

We found that under the conditions used, C-terminal fusions had higher mVenus fluorescence than N-terminal fusions (Figure 5). This phenomenon appeared to be independent of the receptor examined. Despite this, both N-terminal and C-terminal fusions had fluorescence outputs that were significantly above background fluorescence; this finding was consistent between HEK293S and Cos7 cells (Figure 5), and was apparent in live, unfixed cells, as well as cells fixed with PFA (Figure 6). Relative to intracellular fluorescent proteins, extracellular proteins may additionally be more sensitive to quenching or photobleaching due to differences in their environment such as pH. Additionally, we only tested one fusion approach (V_1–154_ was always attached to the receptor, V_155–238_ to RAMP1). This approach was chosen based on previous literature [15], and to provide proof-of-concept for the N-terminal fusion orientation. We did not investigate the alternative combination (V_155–238_ attached to CLR, V_1–154_ attached to RAMP1) and it is possible that alternative positioning or alternative linker sequences may allow for further optimization.

Given our results with these GPCR/RAMP complexes, this N-terminal fusion methodology could potentially be used for investigating the functional trafficking consequences of GPCR/RAMP interactions [48], such as the proposed Glucagon receptor/RAMP2 [49] or parathyroid 1 receptor/RAMP2 complexes [50], or GPCR:GPCR interactions such as the glucagon receptor:glucagon-like peptide 1 receptor complex [51]. This methodology may also be useful for investigating complexes involving RTKs, where C-terminal fusion may interfere with the function of intracellular tyrosine kinase domains [52]. However, the lower fluorescence output we observed with N-terminal fusion should be considered as a potential limitation. Further studies optimizing features such as linker lengths, or using different fluorescent proteins, could be considered to mitigate this lower fluorescence output. Importantly, in all experiments using BiFC methodology, it is important to consider the balance between achieving sufficient signal:noise ratios to enable robust detection in each experimental system, and the deleterious effects of attaching large proteins to receptors.

We saw a trend for BiFC signals to be higher for CLR-based receptors relative to CTR-based receptors (Figure 5). The obligate dimer status of CLR/RAMP1 required for cell surface expression means that every CLR is probably paired with a RAMP; this contrasts with CTR which is able to translocate to the cell surface without RAMP co-expression. Hence, each transfected cell probably expresses a mixed population of free CTR and CTR/RAMP1 complexes on the cell surface [11,26]. As such, the CLR/RAMP1 complex may occur more frequently than the CTR/RAMP1 complex, leading to a higher fluorescence output with CLR-based receptors; however, at this point, this conclusion is purely speculative. Adding to this, the CLR construct used in this study has the native CLR signal sequence replaced with the T8 signal sequence, while the CTR construct uses the native CTR signal sequence. The T8 signal sequence is thought to help drive the associated protein to the cell surface [53], and thus there may be more CLR than CTR directed to the cell surface. Pharmacological experiments comparing the native CLR signal sequence with the T8 signal sequence did not report a difference in pharmacology [13]; however, the cell-surface expression of HA-tagged CLR receptors with and without native signal sequences has not been compared, to our knowledge .

In addition to our findings regarding BiFC positioning, we also show dimerization of CTR/RAMP1 using a BiFC approach for the first time. The heterodimerization of these proteins is accepted based on pharmacological [11], structural [54], co-immunoprecipitation [33], and antibody [55] based experiments; we report here an additional method confirming this interaction. The existence of CTR-CTR homodimers is also reported, with homodimerization proposed to modulate receptor sensitivity to ligand activation [42,56]. Splice variant based homodimerization of CTR has also been proposed to regulate cell surface expression of the receptor [57]. Similarly, the CLR/RAMP1 complex has been proposed to have a 2:1 stoichiometry [15], suggesting that homodimerization could be functionally important, although recent cryo-EM work would suggest that a 1:1 ratio of receptor/RAMP is sufficient for ligand binding [40,58–60]. Multiplexed BiFC based approaches incorporating both N-terminal and C-terminal fusion of two spectrally distinct fluorescent proteins could offer some insight into the relationship between receptor homodimerization, receptor/RAMP heterodimerization, and/or the potential for higher order receptor/RAMP oligomers.

In conclusion, we report that constructs in which BiFC components are fused to the extracellular N-termini of class B GPCRs can create a functional, fluorescent mVenus protein. Under the conditions used with these receptors, the intensity of mVenus signal with N-terminal fusion was lower than that of C-terminal fusion, but N-terminal fusion was better tolerated with regards to cell signaling and ligand-dependent internalization. This increases the versatility of BiFC methodology, allowing further insights into the dimerization and oligomerization of membrane proteins.

## Supplementary Material

Supplementary Figures S1-S9 and Table S1

## Data Availability

All data associated with this manuscript are included with the file. We will make data sets available upon reasonable request from bona fide researchers without undue delay or qualifications.

## Open Access

Open access for this article was enabled by the participation of University of Otago in an all-inclusive *Read & Publish* agreement with Portland Press and the Biochemical Society under a transformative agreement with CAUL.

## Abbreviations

AMY: amylin receptor
ANOVA: analysis of variance
CGRP: calcitonin gene-related peptide
CLR: calcitonin receptor-like receptor
CTR: calcitonin receptor
GPCR: G protein-coupled receptor
PFA: paraformaldehyde
RAMP: receptor activity-modifying protein
RTK: receptor tyrosine kinase
