# Supplementary material for: Extracellular bimolecular fluorescence complementation for investigating membrane protein dimerization: a proof of concept using class B GPCRs
Source: Biosci Rep. 2024 Oct 23;44(10):BSR20240449. doi: 10.1042/BSR20240449 (PMC11499381; doi:10.1042/BSR20240449)
Supplement: Supplementary Figures S1-S9 and Table S1 [file BSR-2024-0449_supp.pdf]

Extracellular bimolecular fluorescence complementation for investigating membrane protein dimerization: a proof of concept using class B GPCRs

Michael L Garelja[1,2,3], Tyla Alexander[1,3], Christopher S Walker[2,3], Debbie L Hay[1,3].*

1. Department of Pharmacology and Toxicology, University of Otago, Dunedin, 9016, New Zealand.

2. School of Biological Sciences, University of Auckland, Auckland, 1010, New Zealand.

3. Maurice Wilkins Centre for Molecular Biodiscovery, University of Auckland, Auckland, New Zealand.

*Authors to whom correspondence should be addressed; michael.garelja@otago.ac.nz, debbie.hay@otago.ac.nz

Contents:

DNA and amino acid sequences of generated constructs p 1-8

List of oligonucleotides used to generate constructs p 9

Supplementary Figures & Table p 10 - 23

WT Receptor Constructs

HA-CLR

T8 Signal Sequence – HA tag – Tag Linker– CLR

DNA

```
atggccttaccagtgaccgccttgctcctgccgctagccttgctgctccacgccgccaggccggatta
cgcgtcttacccgtatgacgtcccagattacgcatcgctgggaggcccttcactcgagggatccgcag
aattagaagagagtcctgaggactcaattcagttgggagttactagaaataaaatcatgacagctcaa
tatgaatgttaccaaaagattatgcaagaccccattcaacaagcagaaggcgtttactgcaacagaac
ctgggatggatggctctgctggaacgatgttgcagcaggaactgaatcaatgcagctctgccctgatt
actttcaggactttgatccatcagaaaaagttacaaagatctgtgaccaagatggaaactggtttaga
catccagcaagcaacagaacatggacaaattatacccagtgtaatgttaacacccacgagaaagtgaa
gactgcactaaatttgttttacctgaccataattggacacggattgtctattgcatcactgcttatct
cgcttggcatattcttttatttcaagagcctaagttgccaaaggattaccttacacaaaaatctgttc
ttctcatttgtttgtaactctgttgtaacaatcattcacctcactgcagtggccaacaaccaggcctt
agtagccacaaatcctgttagttgcaaagtgtcccagttcattcatctttacctgatgggctgtaatt
acttttggatgctctgtgaaggcatttacctacacacactcattgtggtggccgtgtttgcagagaag
caacatttaatgtggtattattttcttggctggggatttccactgattcctgcttgtatacatgccat
tgctagaagcttatattacaatgacaattgctggatcagttctgatacccatctcctctacattatcc
atggcccaatttgtgctgctttactggtgaatctttttttcttgttaaatattgtacgcgttctcatc
accaagttaaaagttacacaccaagcggaatccaatctgtacatgaaagctgtgagagctactcttat
cttggtgccattgcttggcattgaatttgtgctgattccatggcgacctgaaggaaagattgcagagg
aggtatatgactacatcatgcacatccttatgcacttccagggtcttttggtctctaccatttctgc
ttctttaatggagaggttcaagcaattctgagaagaaactggaatcaatacaaaatccaatttggaaa
cagcttttccaactcagaagctcttcgtagtgcgtcttacacagtgtcaacaatcagtgatggtccag
```

gttatagtcatgactgtcctagtgaacacttaaatggaaaagcatccatgatattgaaaatgttctc
ttaaaaccagaaaatttatataattga

Protein

MALPVTALLLPLALLLHAARPDYASYPYDVPDYASLGGPSLEGSAELEESPEDSIQLGVTRNKIMTAQ
YECYQKIMQDPIQQAEGVYCNRTWDGWLCWNDVAAGTESMQLCPDYFQDFDPSEKVTKICDQDGNWFR
HPASNRTWTNYTQCNVNTHEKVKTALNLFYLTIIGHGLSIASLLISLGIFFYFKSLSCQRITLHKNLF
FSFVCNSVVTIIHLTAVANNQALVATNPVSCKVSQFIHLYLMGCNYFWMLCEGIYLHTLIVVAVFAEK
QHLMWYYFLGWGFPLIPACIHAIARSLYYNDNCWISSDTHLLYIIHGPICAALLVNLFFLLNIVRVLI
TKLKVTHQAESNLYMKAVRATLILVPLLGIEFVLIPWRPEGKIAEEVYDYIMHILMHFQGLLVSTIFC
FFNGEVQAILRRNWNQYKIQFGNSFSNSEALRSASYTVSTISDGPGYSHDCPSEHLNGKSIHDIENVL
LKPENLYN

HA-CTR

CTR Signal Sequence – HA tag - CTR

DNA

atgaggttcacatttacaagccggtgcttggcactgtttcttcttctaaatcacccaaccccaattct
tcctgccttttcaaatcaatacccatacgatgttccagattacgctacctatccaacaatagagccca
agccatttctttacgtcgtaggacgaaagaagatgatggatgcacagtacaaatgctatgaccgaatg
cagcagttacccgcataccaaggagaaggtccatattgcaatcgcacctgggatggatggctgtgctg
ggatgacacaccggctggagtattgtcctatcagttctgcccagattattttccggattttgatccat
cagaaaaggttacaaaatactgtgatgaaaaaggtgtttggtttaaacatcctgaaaacaatcgaacc
tggtccaactatactatgtgcaatgctttcactcctgagaaactgaagaatgcatatgttctgtacta
tttggctattgtgggtcattctttgtcaattttcaccctagtgatttccctggggattttcgtgtttt
tcaggagccttggctgccaaagggtaaccctgcacaagaacatgtttcttacttacattctgaattct
atgattatcatcatccacctggttgaagtagtacccaatggagagctcgtgcgaagggacccggtgag
ctgcaagattttgcatttttccaccagtacatgatggcctgcaactatttctggatgctctgtgaag
ggatctatcttcatacactcattgtcgtggctgtgtttactgagaagcaacgcttgcggtggtattat
ctcttgggctggggttcccgctggtgccaaccactatccatgctattaccagggccgtgtacttcaa
tgacaactgctggctgagtgtggaaacccatttgctttacataatccatggacctgtcatggcggcac
ttgtggtcaatttcttctttttgctcaacattgtccgggtgcttgtgaccaaaatgagggaaacccat
gaggcggaatcccacatgtacctgaaggctgtgaaggccaccatgatccttgtgcccctgctgggaat
ccagtttgtcgtctttccctggagaccttccaacaagatgcttgggaagatatatgattacgtgatgc
actctctgattcatttccagggcttctttgttgcgaccatctactgcttctgcaacaatgaggtccaa
accaccgtgaagcgccaatgggcccaattcaaaattcagtggaaccagcgttggggggaggcgcccctc
caaccgctctgctcgcgctgcagccgctgctgcggaggctggcgacatcccaatttacatctgccatc
aggagctgaggaatgaaccagccaacaaccaaggcgaggagagtgctgagatcatccctttgaatatc
atagagcaagagtcatctgct

Protein

MRFTFTSRCLALFLLLNHPTPILPAFSNQYPYDVPDYATYPTIEPKPFLYVVGRKKMMDAQYKCYDRM
QQLPAYQGEGPYCNRTWDGWLCWDDTPAGVLSYQFCPDYFPDFDPSEKVTKYCDEKGVWFKHPENNRT
WSNYTMCNAFTPEKLKNAYVLYYLAIVGHSLSIFTLVISLGIFVFFRSLGCQRVTLHKNMFLTYILNS
MIIIIHLVEVVPNGELVRRDPVSCKILHFFHQYMMACNYFWMLCEGIYLHTLIVVAVFTEKQRLRWYY
LLGWGFPLVPTTIHAITRAVYFNDNCWLSVETHLLYIIHGPVMAALVVNFFFLLNIVRVLVTKMRETH
EAESHMYLKAVKATMILVPLLGIQFVVFPWRPSNKMLGKIYDYVMHSLIHFQGFFVATIYCFCNNEVQ
TTVKRQWAQFKIQWNQRWGRRPSNRSARAAAAAAEAGDIPIYICHQELRNEPANNQGEESAEIIPLNI
IEQESSA

myc-RAMP1

CD33 Signal Sequence – myc tag – Tag Linker – RAMP1

DNA

atgccgctgctgctactgctgcccctgctgtgggcaggggccctggccatggagcaaaagctcatttc
tgaagaggacttgttgcatggatcctgccaggaggctaactacggtgccctcctccgggagctctgcc
tcacccagttccaggtagacatggaggccgtcggggagacgctgtggtgtgactggggcaggaccatc

aggagctacagggagctggccgactgcacctggcacatggcggagaagctgggctgcttctggcccaa
tgcagaggtggacaggttcttcctggcagtgcatggccgctacttcaggagctgccccatctcaggca
gggccgtgcgggacccgcccggcagcatcctctaccccttcatcgtggtccccatcacggtgaccctg
ctggtgacggcactggtggtctggcagagcaagcgcactgagggcattgtg

Protein

MPLLLLLPLLWAGALAMEQKLISEEDLLHGSCQEANYGALLRELCLTQFQVDMEAVGETLWCDWGRTI
RSYRELADCTWHMAEKLGCFWPNAEVDRFFLAVHGRYFRSCPISGRAVRDPPGSILYPFIVVPITVTL
LVTALVVWQSKRTEGIV

C-terminal fusions

C-V_{1-154} gBlock

Venus linker – C-V_{1-154}

DNA

gtgccagttaatagtggcggaggtgggtccatggtcagtaagggtgaggaactctttaccggtgtcgt
ccctattcttgtggaactggatggggacgtaaatgggcacaagttcagtgtttcaggggagggggagg
gtgatgcaacttacggaagctgacgctcaaactcatctgtaccaccggtaaacttccagtcccatgg
ccgactttggtaactaccctcggatacggtctgcaatgttttgccagatacccagaccacatgaagca
acacgactttttaaaagtgctatgcccgagggatatgtacaggaacgcacgattttcttcaaagacg
acggcaactataaaactagggccgaagtgaaatttgagggagatactctcgtcaaccgaatagagttg
aaaggaatagattttaaggaagacggtaacatcctcggacacaagctggaatataactataacagtca
caatgtctatctgaccgcc

Protein

VPVNSGGGGSMVSKGEELFTGVVPILVELDGDVNGHKFSVSGEGEGDATYGKLTLKLICTTGKLPVPW
PTLVTTLGYGLQCFARYPDHMKQHDFFKSAMPEGYVQERTIFFKDDGNYKTRAEVKFEGDTLVNRIEL
KGIDFKEDGNILGHKLEYNYNSHNVYLTA

C-$V_{155-238}$ gBlock

Venus linker – C-$V_{155-238}$

DNA

gtgccagtcaatagcggcggagggggctcagataaacaaaagaacggcatcaaggcgaacttcaagat
aagacacaacatcgaagacggtggcgtgcaacttgccgatcattaccagcagaatacgccgatcgggg
atgggccagtattgctcccggacaatcactacctttcctaccaaagtaaactctccaaagatcccaat
gagaaaagggatcacatggtactcctcgagtttgtcactgcggcggggattacccttggtatggatga
attgtataag

Protein

VPVNSGGGGSDKQKNGIKANFKIRHNIEDGGVQLADHYQQNTPIGDGPVLLPDNHYLSYQSKLSKDPN
EKRDHMVLLEFVTAAGITLGMDELYK

HA-CLR-C-V_{1-154}

T8 Signal Sequence – HA tag – Tag Linker– CLR – Venus Linker – C-V_{1-154}

DNA

atggccttaccagtgaccgccttgctcctgccgctagccttgctgctccacgccgccaggccggatta
cgcgtcttacccgtatgacgtcccagattacgcatcgctgggaggcccttcactcgagggatccgcag
aattagaagagagtcctgaggactcaattcagttgggagttactagaaataaaatcatgacagctcaa
tatgaatgttaccaaaagattatgcaagaccccattcaacaagcagaaggcgtttactgcaacagaac
ctgggatggatggctctgctggaacgatgttgcagcaggaactgaatcaatgcagctctgccctgatt
actttcaggactttgatccatcagaaaaagttacaaagatctgtgaccaagatggaaactggtttaga
catccagcaagcaacagaacatggacaaattatacccagtgtaatgttaacacccacgagaaagtgaa
gactgcactaaatttgttttacctgaccataattggacacggattgtctattgcatcactgcttatct
cgcttggcatattcttttatttcaagagcctaagttgccaaaggattaccttacacaaaaatctgttc

ttctcatttgtttgtaactctgttgtaacaatcattcacctcactgcagtggccaacaaccaggcctt
agtagccacaaatcctgttagttgcaaagtgtcccagttcattcatctttacctgatgggctgtaatt
actttggatgctctgtgaaggcatttacctacacacactcattgtggtggccgtgtttgcagagaag
caacatttaatgtggtattattttcttggctggggatttccactgattcctgcttgtatacatgccat
tgctagaagcttatattacaatgacaattgctggatcagttctgatacccatctcctctacattatcc
atggcccaatttgtgctgctttactggtgaatctttttttcttgttaaatattgtacgcgttctcatc
accaagttaaaagttacacaccaagcggaatccaatctgtacatgaaagctgtgagagctactcttat
cttggtgccattgcttggcattgaatttgtgctgattccatggcgacctgaaggaaagattgcagagg
aggtatatgactacatcatgcacatccttatgcacttccagggtcttttggtctctaccatttctgc
ttctttaatggagaggttcaagcaattctgagaagaaactggaatcaatacaaaatccaatttggaaa
cagcttttccaactcagaagctcttcgtagtgcgtcttacacagtgtcaacaatcagtgatggtccag
gttatagtcatgactgtcctagtgaacacttaaatggaaaaagcatccatgatattgaaaatgttctc
ttaaaaccagaaaatttatataatgtgccagttaatagtggcggaggtgggtccatggtcagtaaggg
tgaggaactctttaccggtgtcgtccctattcttgtggaactggatggggacgtaaatgggcacaagt
tcagtgtttcaggggagggggagggtgatgcaacttacggaagctgacgctcaaactcatctgtacc
accggtaaacttccagtcccatggccgactttggtaactaccctcggatacggtctgcaatgttttgc
cagatacccagaccacatgaagcaacacgactttttaaaagtgctatgcccgagggatatgtacagg
aacgcacgatttcttcaaagacgacggcaactataaaactagggccgaagtgaaatttgagggagat
actctcgtcaaccgaatagagttgaaaggaatagatttaaggaagacggtaacatcctcggacacaa
gctggaatataactataacagtcacaatgtctatctgaccgcc

Protein

MALPVTALLLPLALLLHAARPDYASYPYDVPDYASLGGPSLEGSAELEESPEDSIQLGVTRNKIMTAQ
YECYQKIMQDPIQQAEGVYCNRTWDGWLCWNDVAAGTESMQLCPDYFQDFDPSEKVTKICDQDGNWFR
HPASNRTWTNYTQCNVNTHEKVKTALNLFYLTIIGHGLSIASLLISLGIFFYFKSLSCQRITLHKNLF
FSFVCNSVVTIIHLTAVANNQALVATNPVSCKVSQFIHLYLMGCNYFWMLCEGIYLHTLIVVAVFAEK
QHLMWYYFLGWGFPLIPACIHAIARSLYYNDNCWISSDTHLLYIIHGPICAALLVNLFFLLNIVRVLI
TKLKVTHQAESNLYMKAVRATLILVPLLGIEFVLIPWRPEGKIAEEVYDYIMHILMHFQGLLVSTIFC
FFNGEVQAILRRNWNQYKIQFGNSFSNSEALRSASYTVSTISDGPGYSHDCPSEHLNGKSIHDIENVL
LKPENLYNVPVNSGGGGSMVSKGEELFTGVVPILVELDGDVNGHKFSVSGEGEGDATYGKLTLKLICT
TGKLPVPWPTLVTTLGYGLQCFARYPDHMKQHDFFKSAMPEGYVQERTIFFKDDGNYKTRAEVKFEGD
TLVNRIELKGIDFKEDGNILGHKLEYNYNSHNVYLTA

HA-CTR-C-V_{1-154}

Signal Sequence – HA tag– CTR – Venus Linker – C-V_{1-154}

DNA

atgaggttcacatttacaagccggtgcttggcactgtttcttcttctaaatcacccaaccccaattct
tcctgccttttcaaatcaatacccatacgatgttccagattacgctacctatccaacaatagagccca
agccatttctttacgtcgtaggacgaaagaagatgatggatgcacagtacaaatgctatgaccgaatg
cagcagttacccgcataccaaggagaaggtccatattgcaatcgcacctgggatggatggctgtgctg
ggatgacacaccggctggagtattgtcctatcagttctgcccagattattttccggatttgatccat
cagaaaaggttacaaaatactgtgatgaaaaaggtgtttggtttaaacatcctgaaaacaatcgaacc
tggtccaactatactatgtgcaatgctttcactcctgagaaactgaagaatgcatatgttctgtacta
tttggctattgtgggtcattctttgtcaattttcaccctagtgatttccctggggattttcgtgtttt
tcaggagccttggctgccaaagggtaaccctgcacaagaacatgtttcttacttacattctgaattct
atgattatcatcatccacctggttgaagtagtacccaatggagagctcgtgcgaagggacccggtgag
ctgcaagatttgcatttttttccaccagtacatgatggcctgcaactatttctggatgctctgtgaag
ggatctatcttcatacactcattgtcgtggctgtgtttactgagaagcaacgcttgcggtggtattat
ctcttgggctgggggttcccgctggtgccaaccactatccatgctattaccagggccgtgtacttcaa
tgacaactgctggctgagtgtggaaacccatttgctttacataatccatggacctgtcatggcggcac
ttgtggtcaatttcttcttttgctcaacattgtccgggtgcttgtgaccaaaatgagggaaacccat
gaggcggaatcccacatgtacctgaaggctgtgaaggccaccatgatccttgtgcccctgctgggaat
ccagtttgtcgtctttccctggagaccttccaacaagatgcttgggaagatatatgattacgtgatgc
actctctgattcatttccagggcttctttgttgcgaccatctactgcttctgcaacaatgaggtccaa
accaccgtgaagcgccaatgggcccaattcaaaattcagtggaaccagcgttgggggaggcgcccctc

caaccgctctgctcgcgctgcagccgctgctgcggaggctggcgacatcccaatttacatctgccatc
aggagctgaggaatgaaccagccaacaaccaaggcgaggagagtgctgagatcatccctttgaatatc
atagagcaagagtcatctgctgtgccagttaatagtggcggaggtgggtccatggtcagtaagggtga
ggaactctttaccggtgtcgtccctattcttgtggaactggatggggacgtaaatgggcacaagttca
gtgtttcaggggaggggagggtgatgcaacttacggaagctgacgctcaaactcatctgtaccacc
ggtaaacttccagtcccatggccgactttggtaactaccctcggatacggtctgcaatgttttgccag
atacccagaccacatgaagcaacacgactttttaaaagtgctatgcccgagggatatgtacaggaac
gcacgatttcttcaaagacgacggcaactataaaactagggccgaagtgaaatttgagggagatact
ctcgtcaaccgaatagagttgaaaggaatagattttaaggaagacggtaacatcctcggacacaagct
ggaatataactataacagtcacaatgtctatctgaccgcc

Protein

MRFTFTSRCLALFLLLNHPTPILPAFSNQYPYDVPDYATYPTIEPKPFLYVVGRKKMMDAQYKCYDRM
QQLPAYQGEGPYCNRTWDGWLCWDDTPAGVLSYQFCPDYFPDFDPSEKVTKYCDEKGVWFKHPENNRT
WSNYTMCNAFTPEKLKNAYVLYYLAIVGHSLSIFTLVISLGIFVFFRSLGCQRVTLHKNMFLTYILNS
MIIIIHLVEVVPNGELVRRDPVSCKILHFFHQYMMACNYFWMLCEGIYLHTLIVVAVFTEKQRLRWYY
LLGWGFPLVPTTIHAITRAVYFNDNCWLSVETHLLYIIHGPVMAALVVNFFFLLNIVRVLVTKMRETH
EAESHMYLKAVKATMILVPLLGIQFVVFPWRPSNKMLGKIYDYVMHSLIHFQGFFVATIYCFCNNEVQ
TTVKRQWAQFKIQWNQRWGRRPSNRSARAAAAAAEAGDIPIYICHQELRNEPANNQGEESAEIIPLNI
IEQESSAVPVNSGGGGSMVSKGEELFTGVVPILVELDGDVNGHKFSVSGEGEGDATYGKLTLKLICTT
GKLPVPWPTLVTTLGYGLQCFARYPDHMKQHDFFKSAMPEGYVQERTIFFKDDGNYKTRAEVKFEGDT
LVNRIELKGIDFKEDGNILGHKLEYNYNSHNVYLTA

RAMP1-C-$V_{155\text{-}238}$

CD33 Signal Sequence – myc tag – Tag linker – RAMP1 – Venus Linker – C-$V_{155\text{-}238}$

DNA

atgccgctgctgctactgctgcccctgctgtgggcaggggccctggccatggagcaaaagctcatttc
tgaagaggacttgttgcatggatcctgccaggaggctaactacggtgccctcctccgggagctctgcc
tcacccagttccaggtagacatggaggccgtcggggagacgctgtggtgtgactggggcaggaccatc
aggagctacagggagctggccgactgcacctggcacatggcggagaagctgggctgcttctggcccaa
tgcagaggtggacaggttcttcctggcagtgcatggccgctacttcaggagctgccccatctcaggca
gggccgtgcgggacccgcccggcagcatcctctaccccttcatcgtggtccccatcacggtgaccctg
ctggtgacggcactggtggtctggcagagcaagcgcactgagggcattgtggtgccagtcaatagcgg
cggagggggctcagataaacaaaagaacggcatcaaggcgaacttcaagataagacacaacatcgaag
acggtggcgtgcaacttgccgatcattaccagcagaatacgccgatcggggatgggccagtattgctc
ccggacaatcactacctttcctaccaaagtaaactctccaaagatcccaatgagaaaaggatcacat
ggtactcctcgagtttgtcactgcggcggggattacccttggtatggatgaattgtataag

Protein

MPLLLLLPLLWAGALAMEQKLISEEDLLHGSCQEANYGALLRELCLTQFQVDMEAVGETLWCDWGRTI
RSYRELADCTWHMAEKLGCFWPNAEVDRFFLAVHGRYFRSCPISGRAVRDPPGSILYPFIVVPITVTL
LVTALVVWQSKRTEGIVVPVNSGGGGSDKQKNGIKANFKIRHNIEDGGVQLADHYQQNTPIGDGPVLL
PDNHYLSYQSKLSKDPNEKRDHMVLLEFVTAAGITLGMDELYK

N-terminal tags

N-$V_{1\text{-}154}$ gBlock

N-$V_{1\text{-}154}$ – Venus Linker

DNA

atggtcagtaagggagaggaactcttcacaggtgtggtgccaatcttggtagagctggacggcgacgt
caatggtcacaagtttagcgtgtccggtgaaggggagggagatgcaacctatggaaagctcactctta
aatttatatgcaccacgggaaagctcccggttccgtggcccaccctggtgaccacgttcggctacggg
ctccaatgtttcgccaggtatccagatcatatgaagcagcacgactttttcaaatccgctatgccgga
ggggtatgtacaagagagaacaatttttttcaaggatgatggcaattacaagacgagagcagaggtga

aatttgaaggggacactctggttaatcgaattgaactcaaaggcattgactttaaagaggacggtaac
attcttgggcataaactggaatacaactacaactctcacaacgtatatataatggccgggtctgccgg
tagtgccggatcagcg

Protein

MVSKGEELFTGVVPILVELDGDVNGHKFSVSGEGEGDATYGKLTLKFICTTGKLPVPWPTLVTTFGYG
LQCFARYPDHMKQHDFFKSAMPEGYVQERTIFFKDDGNYKTRAEVKFEGDTLVNRIELKGIDFKEDGN
ILGHKLEYNYNSHNVYIMAGSAGSAGSA

N-$V_{155-238}$ gBlock

N-$V_{155-238}$ – Venus Linker

DNA

gacaaacagaaaaatggaattaaagcgaacttcaaaatccgacacaacattgaagacggtggtgttca
actcgcggaccattatcaacaaaacactccgataggtgacggccctgtgctgttgcccgacaaccatt
atctgtcctatcaaagcaaactttccaaggaccccaatgaaaaacgcgatcatatggtactcctggag
ttcgtgacggccgcaggtatcacgttgggtatggatgaactctacaaaggatctgcagggtcagcggg
ctccgcg

Protein

DKQKNGIKANFKIRHNIEDGGVQLADHYQQNTPIGDGPVLLPDNHYLSYQSKLSKDPNEKRDHMVLLE
FVTAAGITLGMDELYKGSAGSAGSA

N-V_{1-154}-CLR

T8 Signal Sequence – HA tag – Tag Linker – N-V_{1-154} – Venus Linker –CLR

DNA

atggccttaccagtgaccgccttgctcctgccgctagccttgctgctccacgccgccaggccggatta
cgcgtcttacccgtatgacgtcccagattacgcatcgctgggaggcccttcactcgagggatccgca
atggtcagtaagggagaggaactcttcacaggtgtggtgccaatcttggtagagctggacggcgacgt
caatggtcacaagtttagcgtgtccggtgaaggggagggagatgcaacctatggaaagctcactctta
aatttatatgcaccacgggaaagctcccggttccgtggcccaccctggtgaccacgttcggctacggg
ctccaatgtttcgccaggtatccagatcatatgaagcagcacgacttttttcaaatccgctatgccgga
ggggtatgtacaagagagaacaatttttttcaaggatgatggcaattacaagacgagagcagaggtga
aatttgaaggggacactctggttaatcgaattgaactcaaaggcattgactttaaagaggacggtaac
attcttgggcataaactggaatacaactacaactctcacaacgtatatataatggccgggtctgccgg
tagtgccggatcagcggaattagaagagagtcctgaggactcaattcagttgggagttactagaaata
aaatcatgacagctcaatatgaatgttaccaaaagattatgcaagaccccattcaacaagcagaaggc
gtttactgcaacagaacctgggatggatggctctgctggaacgatgttgcagcaggaactgaatcaat
gcagctctgccctgattactttcaggactttgatccatcagaaaaagttacaaagatctgtgaccaag
atggaaactggtttagacatccagcaagcaacagaacatggacaaattatacccagtgtaatgttaac
acccacgagaaagtgaagactgcactaaatttgttttacctgaccataattggacacggattgtctat
tgcatcactgcttatctcgcttggcatattcttttatttcaagagcctaagttgccaaaggattacct
tacacaaaaatctgttcttctcatttgtttgtaactctgttgtaacaatcattcacctcactgcagtg
gccaacaaccaggccttagtagccacaaatcctgttagttgcaaagtgtcccagttcattcatcttta
cctgatgggctgtaattacttttggatgctctgtgaaggcatttacctacacacactcattgtggtgg
ccgtgtttgcagagaagcaacatttaatgtggtattattttcttggctggggatttccactgattcct
gcttgtatacatgccattgctagaagcttatattacaatgacaattgctggatcagttctgataccca
tctcctctacattatccatggcccaatttgtgctgctttactggtgaatctttttttcttgttaaata
ttgtacgcgttctcatcaccaagttaaaagttacacaccaagcggaatccaatctgtacatgaaagct
gtgagagctactcttatcttggtgccattgcttggcattgaatttgtgctgattccatggcgacctga
aggaaagattgcagaggaggtatatgactacatcatgcacatccttatgcacttccagggtcttttgg
tctctaccatttctgcttctttaatggagaggttcaagcaattctgagaagaaactggaatcaatac
aaaatccaatttggaaacagcttttccaactcagaagctcttcgtagtgcgtcttacacagtgtcaac
aatcagtgatggtccaggttatagtcatgactgtcctagtgaacacttaaatggaaaaagcatccatg
atattgaaaatgttctcttaaaaccagaaaatttatataattga

Protein

MALPVTALLLPLALLLHAARPDYASYPYDVPDYASLGGPSLEGSAMVSKGEELFTGVVPILVELDGDV
NGHKFSVSGEGEGDATYGKLTLKFICTTGKLPVPWPTLVTTFGYGLQCFARYPDHMKQHDFFKSAMPE
GYVQERTIFFKDDGNYKTRAEVKFEGDTLVNRIELKGIDFKEDGNILGHKLEYNYNSHNVYIMAGSAG
SAGSAELEESPEDSIQLGVTRNKIMTAQYECYQKIMQDPIQQAEGVYCNRTWDGWLCWNDVAAGTESM
QLCPDYFQDFDPSEKVTKICDQDGNWFRHPASNRTWTNYTQCNVNTHEKVKTALNLFYLTIIGHGLSI
ASLLISLGIFFYFKSLSCQRITLHKNLFFSFVCNSVVTIIHLTAVANNQALVATNPVSCKVSQFIHLY
LMGCNYFWMLCEGIYLHTLIVVAVFAEKQHLMWYYFLGWGFPLIPACIHAIARSLYYNDNCWISSDTH
LLYIIHGPICAALLVNLFFLLNIVRVLITKLKVTHQAESNLYMKAVRATLILVPLLGIEFVLIPWRPE
GKIAEEVYDYIMHILMHFQGLLVSTIFCFFNGEVQAILRRNWNQYKIQFGNSFSNSEALRSASYTVST
ISDGPGYSHDCPSEHLNGKSIHDIENVLLKPENLYN

N-V$_{1-154}$-CTR

Signal Sequence – N-V$_{1-154}$ – Venus Linker –HA tag –CTR

DNA

atgaggttcacatttacaagccggtgcttggcactgtttcttcttctaaatcacccaaccccaattct
tcctatggtcagtaagggagaggaactcttcacaggtgtggtgccaatcttggtagagctggacggcg
acgtcaatggtcacaagtttagcgtgtccggtgaaggggagggagatgcaacctatggaaagctcact
cttaaatttatatgcaccacgggaaagctcccggttccgtggcccaccctggtgaccacgttcggcta
cgggctccaatgtttcgccaggtatccagatcatatgaagcagcacgactttttcaaatccgctatgc
cggaggggtatgtacaagagagaacaatttttttcaaggatgatggcaattacaagacgagagcagag
gtgaaatttgaaggggacactctggttaatcgaattgaactcaaaggcattgactttaaagaggacgg
taacattcttggcataaactggaatacaactacaactctcacaacgtatatataatggccgggtctg
ccggtagtgccggatcagcggccttttcaaatcaatacccatacgatgttccagattacgctacctat
ccaacaatagagcccaagccatttctttacgtcgtaggacgaaagaagatgatggatgcacagtacaa
atgctatgaccgaatgcagcagttacccgcataccaaggagaaggtccatattgcaatcgcacctggg
atggatggctgtgctgggatgacacaccggctggagtattgtcctatcagttctgcccagattatttt
ccggattttgatccatcagaaaaggttacaaaatactgtgatgaaaaaggtgtttggtttaaacatcc
tgaaaacaatcgaacctggtccaactatactatgtgcaatgctttcactcctgagaaactgaagaatg
catatgttctgtactatttggctattgtgggtcattctttgtcaattttcaccctagtgatttccctg
ggatttcgtgtttttcaggagccttggctgccaaagggtaaccctgcacaagaacatgtttcttac
ttacattctgaattctatgattatcatcatccacctggttgaagtagtacccaatggagagctcgtgc
gaagggacccggtgagctgcaagatttgcatttttccaccagtacatgatggcctgcaactatttc
tggatgctctgtgaagggatctatcttcatacactcattgtcgtggctgtgtttactgagaagcaacg
cttgcggtggtattatctcttgggctggggttcccgctggtgccaaccactatccatgctattacca
gggccgtgtacttcaatgacaactgctggctgagtgtggaaacccatttgctttacataatccatgga
cctgtcatggcggcacttgtggtcaatttcttctttttgctcaacattgtccgggtgcttgtgaccaa
aatgagggaaacccatgaggcggaatcccacatgtacctgaaggctgtgaaggccaccatgatccttg
tgcccctgctgggaatccagtttgtcgtctttccctggagaccttccaacaagatgcttgggaagata
tatgattacgtgatgcactctctgattcatttccaggcttctttgttgcgaccatctactgcttctg
caacaatgaggtccaaaccaccgtgaagcgccaatgggcccaattcaaaattcagtggaaccagcgtt
ggggggaggcgcccctccaaccgctctgctcgcgctgcagccgctgctgcggaggctggcgacatccca
atttacatctgccatcaggagctgaggaatgaaccagccaacaaccaaggcgaggagagtgctgagat
catccctttgaatatcatagagcaagagtcatctgct

Protein

MRFTFTSRCLALFLLLNHPTPILPMVSKGEELFTGVVPILVELDGDVNGHKFSVSGEGEGDATYGKLT
LKFICTTGKLPVPWPTLVTTFGYGLQCFARYPDHMKQHDFFKSAMPEGYVQERTIFFKDDGNYKTRAE
VKFEGDTLVNRIELKGIDFKEDGNILGHKLEYNYNSHNVYIMAGSAGSAGSAAFSNQYPYDVPDYATY
PTIEPKPFLYVVGRKKMMDAQYKCYDRMQQLPAYQGEGPYCNRTWDGWLCWDDTPAGVLSYQFCPDYF
PDFDPSEKVTKYCDEKGVWFKHPENNRTWSNYTMCNAFTPEKLKNAYVLYYLAIVGHSLSIFTLVISL
GIFVFFRSLGCQRVTLHKNMFLTYILNSMIIIHLVEVVPNGELVRRDPVSCKILHFFHQYMMACNYF
WMLCEGIYLHTLIVVAVFTEKQRLRWYYLLGWGFPLVPTTIHAITRAVYFNDNCWLSVETHLLYIIHG
PVMAALVVNFFFLLNIVRVLVTKMRETHEAESHMYLKAVKATMILVPLLGIQFVVFPWRPSNKMLGKI

YDYVMHSLIHFQGFFVATIYCFCNNEVQTTVKRQWAQFKIQWNQRWGRRPSNRSARAAAAAAEAGDIP
IYICHQELRNEPANNQGEESAEIIPLNIIEQESSA

N-$V_{155-238}$-RAMP1

CD33 Signal Sequence – myc tag – Tag Linker – N-$V_{155-238}$ – Venus Linker – RAMP1

DNA

atgccgctgctgctactgctgcccctgctgtgggcaggggccctggccatggagcaaaagctcatttc
tgaagaggacttgttgcatggatccgacaaacagaaaaatggaattaaagcgaacttcaaaatccgac
acaacattgaagacggtggtgttcaactcgcggaccattatcaacaaaacactccgataggtgacggc
cctgtgctgttgcccgacaaccattatctgtcctatcaaagcaaactttccaaggaccccaatgaaaa
acgcgatcatatggtactcctggagttcgtgacggccgcaggtatcacgttgggtatggatgaactct
acaaaggatctgcagggtcagcgggctccgcgtgccaggaggctaactacggtgccctcctccgggag
ctctgcctcacccagttccaggtagacatggaggccgtcggggagacgctgtggtgtgactggggcag
gaccatcaggagctacagggagctggccgactgcacctggcacatggcggagaagctgggctgcttct
ggcccaatgcagaggtggacaggttcttcctggcagtgcatggccgctacttcaggagctgccccatc
tcaggcagggccgtgcgggacccgcccggcagcatcctctaccccttcatcgtggtccccatcacggt
gaccctgctggtgacggcactggtggtctggcagagcaagcgcactgagggcattgtgtag

Protein

MPLLLLLPLLWAGALAMEQKLISEEDLLHGSDKQKNGIKANFKIRHNIEDGGVQLADHYQQNTPIGDG
PVLLPDNHYLSYQSKLSKDPNEKRDHMVLLEFVTAAGITLGMDELYKGSAGSAGSACQEANYGALLRE
LCLTQFQVDMEAVGETLWCDWGRTIRSYRELADCTWHMAEKLGCFWPNAEVDRFFLAVHGRYFRSCPI
SGRAVRDPPGSILYPFIVVPITVTLLVTALVVWQSKRTEGIV

Primer list:

Receptor/RAMP linearization

Target	Insert site	Direction	Sequence	Notes
CLR	C-terminal	Forward	ACAATGTCTATCTGACCGCCTGAGAATTCCACCACACTG	Linearization incorporated overhang for C-V_{1-154} insertion
CLR	C-terminal	Reverse	CCGCCACTATTAACTGGCACATTATATAAATTTTCTGGTTTTAAGAGAAC	Linearization incorporated overhang for C-V_{1-154} insertion
CTR	C-terminal	Forward	TGAGCGGCCGCTCGAGTC	-
CTR	C-terminal	Reverse	AGCAGATGACTCTTGCTCTATG	-
RAMP1	C-terminal	Forward	TAGAATTCGCGGCCGCTCG	-
RAMP1	C-terminal	Reverse	CACAATGCCCTCAGTGCGC	-
CLR	N-terminal	Forward	GAATTAGAAGAGAGTCCTGAGGA	-
CLR	N-terminal	Reverse	TGCGGATCCCTCGAGTGA	-
CTR	N-terminal	Forward	GCCTTTTCAAATCAATACCCATACG	-
CTR	N-terminal	Reverse	AGGAAGAATTGGGGTTGGG	-
RAMP1	N-terminal	Forward	TGCCAGGAGGCTAACTACGG	-
RAMP1	N-terminal	Reverse	GGATCCATGCAACAAGTCC	-

Insert amplification and overhang introduction

gBlock	Target, insert site	Direction	Sequence	Notes
C-V_{1-154}	CLR, C-terminal	Forward	GTGCCAGTTAATAGTGGCGGAGG	Overhang introduced during receptor linearization
C-V_{1-154}	CLR, C-terminal	Reverse	GGCGGTCAGATAGACATTGTGACT	Overhang introduced during receptor linearization
C-V_{1-154}	CTR, C-terminal	Forward	CAAGAGTCATCTGCTGTGCCAGTTAATAGTGGCGGAGG	-
C-V_{1-154}	CTR, C-terminal	Reverse	TCGAGCGGCCGCTCAGGCGGTCAGATAGACATTGTGAC	-
C-$V_{155-238}$	RAMP1, C-terminal	Forward	ACTGAGGGCATTGTGGTGCCAGTCAATAGCGGC	-
C-$V_{155-238}$	RAMP1, C-terminal	Reverse	CGGCCGCGAATTCTACTTATACAATTCATCCATACCAAGG	-
N-V_{1-154}	CLR, N-terminal	Forward	CTCGAGGGATCCGCAATGGTCAGTAAGGGAGAGGAAC	-
N-V_{1-154}	CLR, N-terminal	Reverse	ACTCTCTTCTAATTCCGCTGATCCGGCACTACC	-
N-V_{1-154}	CTR, N-terminal	Forward	ACCCCAATTCTTCCTATGGTCAGTAAGGGAGAGGAAC	-
N-V_{1-154}	CTR, N-terminal	Reverse	TTGATTTGAAAAGGCCGCTGATCCGGCACTACC	-
N-$V_{155-238}$	RAMP1, N-terminal	Forward	TTGTTGCATGGATCCGACAAACAGAAAAATGGAATTAAAG	-
N-$V_{155-238}$	RAMP1, N-terminal	Reverse	GTTAGCCTCCTGGCACGCGGAGCCCGCTGACCC	-

Supplementary Figures

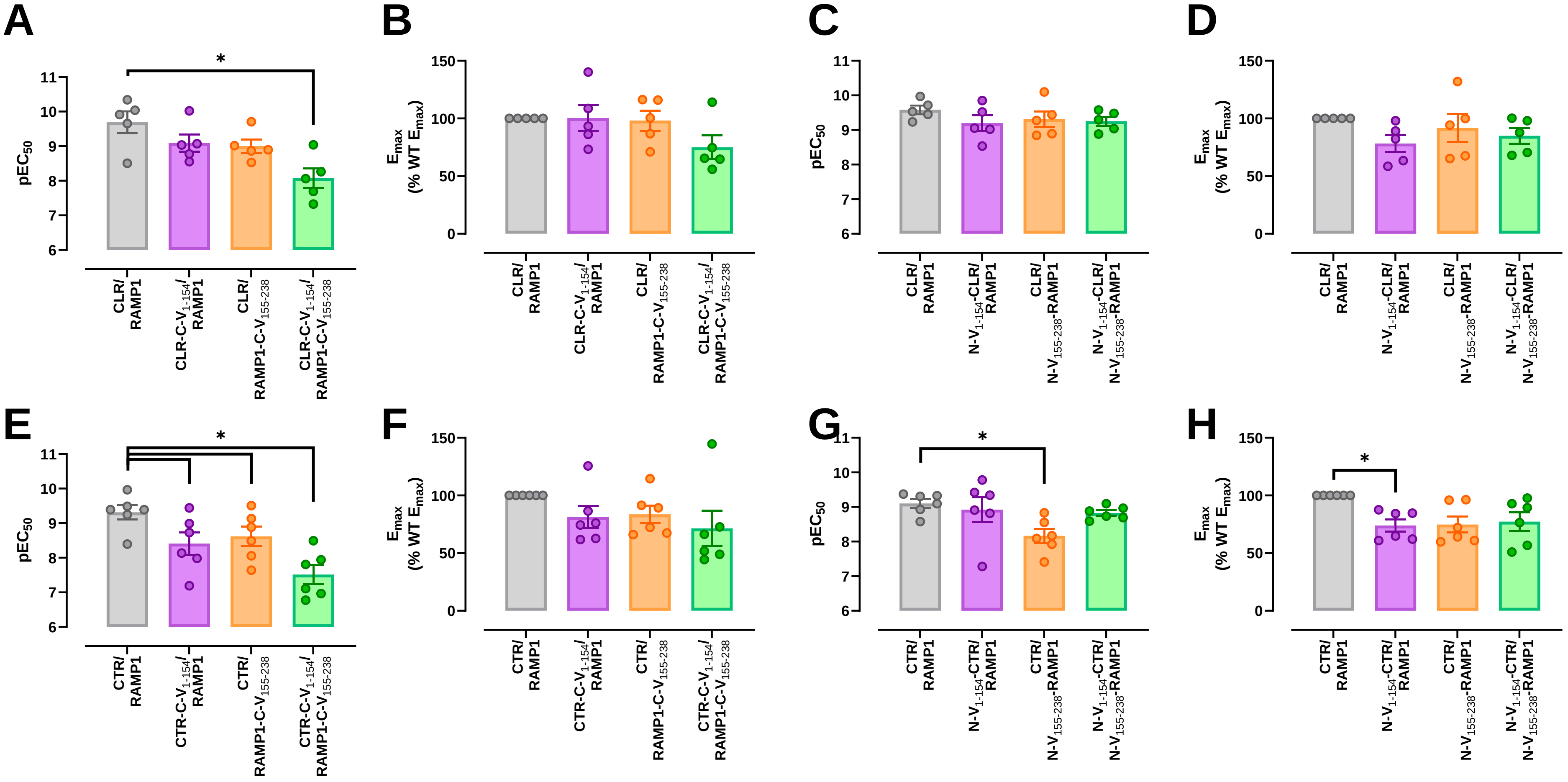

Supplemental Figure 1. Summary of pEC_{50} and E_{max} data from CLR/RAMP1 and CTR/RAMP1 complexes in Cos7. Panels A & B are C-terminal fusion constructs of the CLR/RAMP1 complex. Panels C & D are N-terminal fusion constructs of the CLR/RAMP1 complex. Panels E & F are C-terminal fusion constructs of the CTR/RAMP1 complex. Panels G & H are N-terminal fusion constructs of the CTR/RAMP1 complex. Panels A, C, E, and G are pEC_{50} values, and panels B, D, F, and H are E_{max} values, normalized to the WT condition in each experiment. Each data point is a result from a single experiment, while lines indicate mean ± s.e.m. Data analyzed using repeated measures one-way ANOVA with Dunnet's post-hoc test, comparing each condition to WT. Statistical testing for E_{max} data was performed on log-transformed raw E_{max} values; normalized values presented here for presentation purposes only.

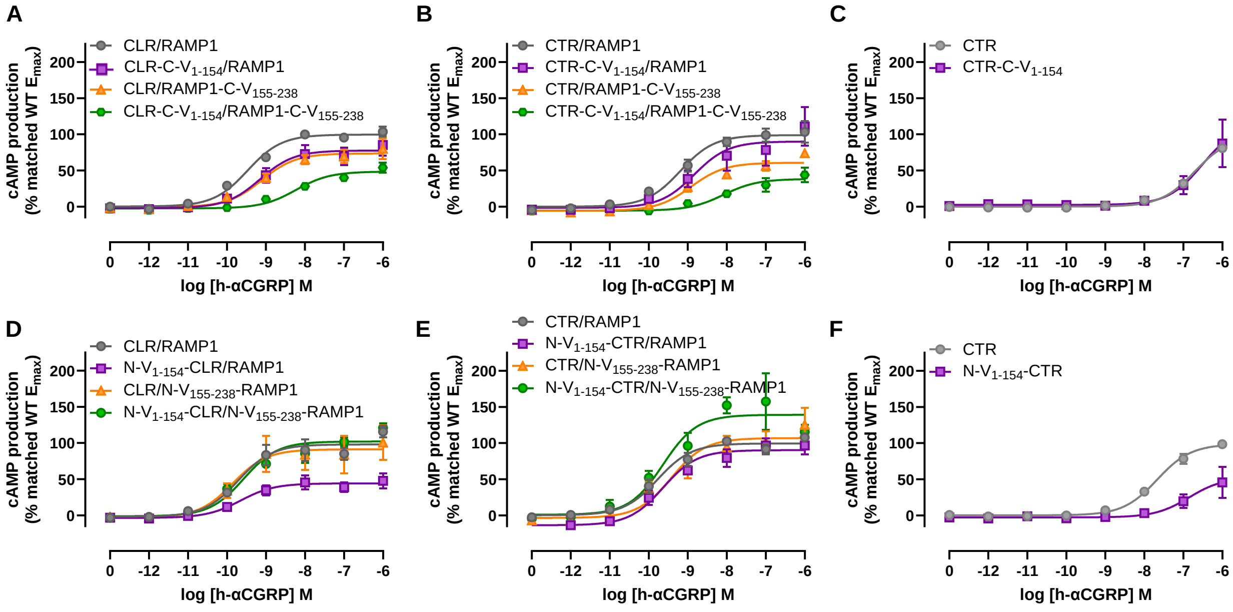
A
CLR/RAMP1
CLR-C-V1-154/RAMP1
CLR/RAMP1-C-V155-238
CLR-C-V1-154/RAMP1-C-V155-238
cAMP production (% matched WT Emax)
log [h-αCGRP] M
B
CTR/RAMP1
CTR-C-V1-154/RAMP1
CTR/RAMP1-C-V155-238
CTR-C-V1-154/RAMP1-C-V155-238
C
CTR
CTR-C-V1-154
D
CLR/RAMP1
N-V1-154-CLR/RAMP1
CLR/N-V155-238-RAMP1
N-V1-154-CLR/N-V155-238-RAMP1
E
CTR/RAMP1
N-V1-154-CTR/RAMP1
CTR/N-V155-238-RAMP1
N-V1-154-CTR/N-V155-238-RAMP1
F
CTR
N-V1-154-CTR

Supplemental Figure 2. Concentration-response curves for human (h-) αCGRP stimulating cAMP production at CGRP or AMY_1 receptors incorporating C-terminal (A, B, C), or N-terminal (D, E, F) mVenus BiFC fusion constructs (15-minute stimulation duration). Experiments were performed in transiently transfected HEK293S cells. Each point is the mean ± s.e.m. of at least 3 independent experiments performed in duplicate or triplicate, for exact *n* numbers see Supplemental Table 1.

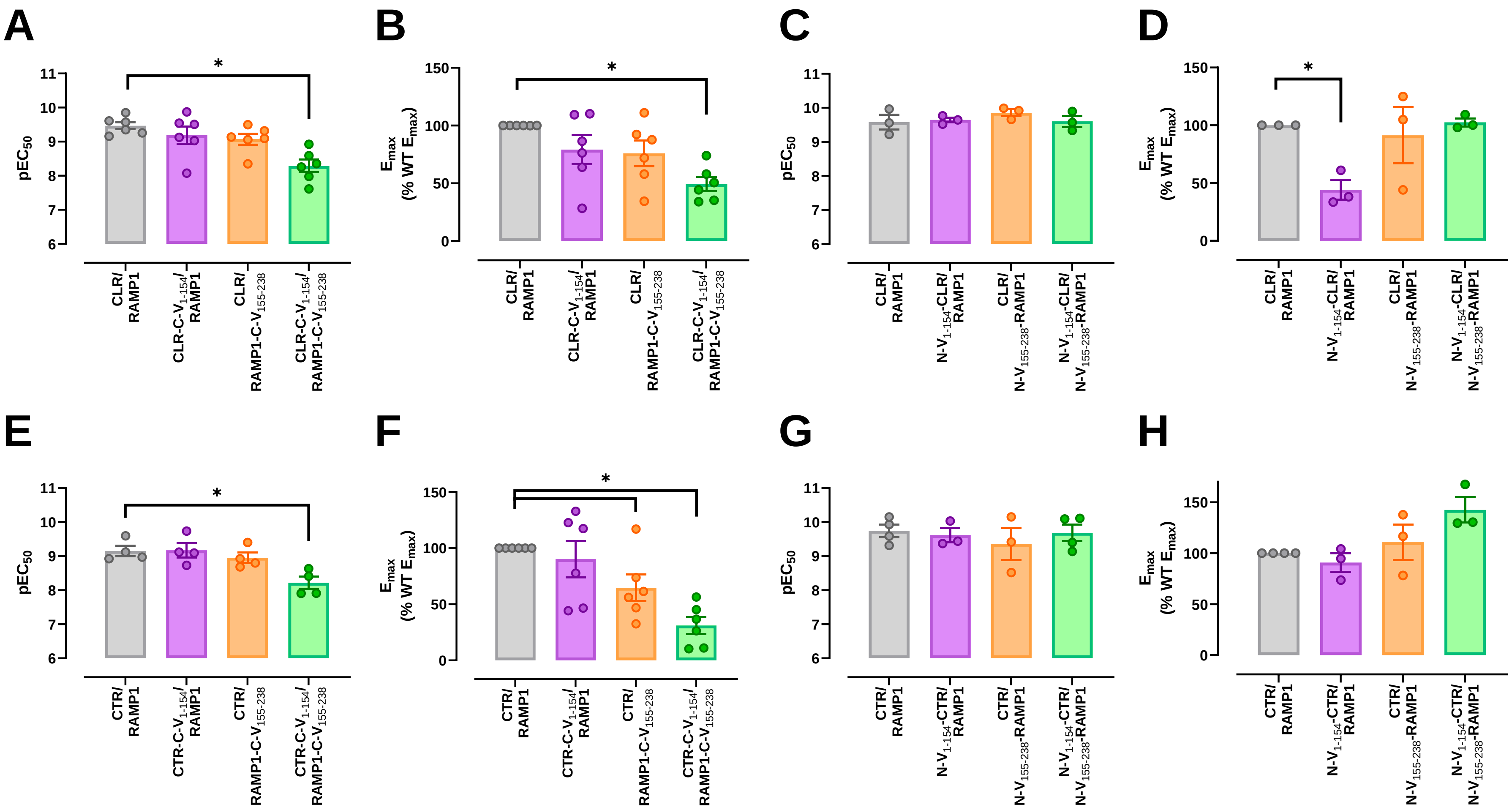
A
B
C
D
E
F
G
H
pEC50
Emax (% WT Emax)
*
CLR/ RAMP1
CLR-C-V1-154/ RAMP1
CLR/ RAMP1-C-V155-238
CLR-C-V1-154/ RAMP1-C-V155-238
N-V1-154-CLR/ RAMP1
CLR/ N-V155-238-RAMP1
N-V1-154-CLR/ N-V155-238-RAMP1
CTR/ RAMP1
CTR-C-V1-154/ RAMP1
CTR/ RAMP1-C-V155-238
CTR-C-V1-154/ RAMP1-C-V155-238
N-V1-154-CTR/ RAMP1
CTR/ N-V155-238-RAMP1
N-V1-154-CTR/ N-V155-238-RAMP1

Supplemental Figure 3. Summary of pEC_{50} and E_{max} data from CLR/RAMP1 and CTR/RAMP1 complexes in HEK293S cells. Panels A & B are C-terminal fusion constructs of the CLR/RAMP1 complex. Panels C & D are N-terminal fusion constructs of the CLR/RAMP1 complex. Panels E & F are C-terminal fusion constructs of the CTR/RAMP1 complex. Panels G & H are N-terminal fusion constructs of the CTR/RAMP1 complex. Panels A, C, E, and G are pEC_{50} values, and panels B, D, F, and H are E_{max} values, normalized to the WT condition in each experiment. Each data point is a result from a single experiment, while lines indicate mean ± s.e.m. Data analyzed using repeated measures one-way ANOVA with Dunnet's post-hoc test, comparing each condition to WT. Statistical testing for E_{max} data was performed on log-transformed raw E_{max} values; normalized values presented here for presentation purposes only.

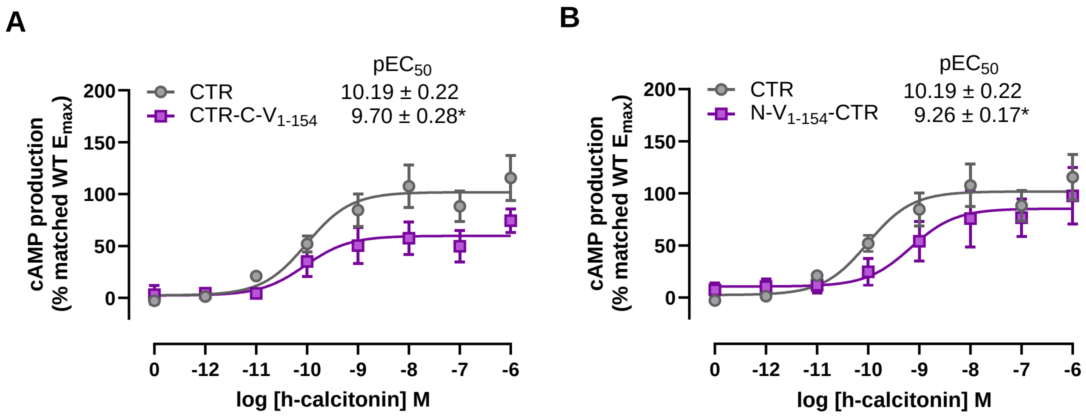
A
pEC50
CTR 10.19 ± 0.22
CTR-C-V1-154 9.70 ± 0.28*
cAMP production (% matched WT Emax)
200
150
100
50
0
0 -12 -11 -10 -9 -8 -7 -6
log [h-calcitonin] M
B
pEC50
CTR 10.19 ± 0.22
N-V1-154-CTR 9.26 ± 0.17*
cAMP production (% matched WT Emax)
200
150
100
50
0
0 -12 -11 -10 -9 -8 -7 -6
log [h-calcitonin] M

Supplemental Figure 4. Concentration-response curves for human (h-) calcitonin stimulating cAMP production at CTR incorporating C-terminal (A), or N-terminal (B) $V_{1\text{-}154}$ mVenus BiFC fusion constructs. Experiments were performed in transiently transfected Cos7 cells using a 15-minute stimulation duration. Each point is the mean ± s.e.m. of 4 independent experiments performed in duplicate. pEC_{50} values as mean ± s.e.m. shown in the figure; pEC_{50} and E_{max} values were statistically compared using repeated measures one-way ANOVA (each experiment included all treatment groups on one plate, and thus data were analysed together, but are separated here for ease of comparison); * indicates a significant difference from CTR. There were no significant differences in E_{max} although there was a trend for a reduced E_{max} with CTR-C-$V_{1\text{-}154}$. The control CTR curve is the same across panels; this is duplicated this for ease of comparison.

Supplemental Table 1. pEC_{50} and E_{max} values for human αCGRP stimulated cAMP production at fusion constructs encoding V_{1-154} and/or $V_{155-238}$ attached to either the N or C terminus of CGRP or AMY_1 receptor subunits in transiently transfected HEK293S cells.

Fusion position	Receptor	WT receptor			V_{1-154} fusion alone			$V_{155-238}$ fusion alone			V_{1-154} and $V_{155-238}$ fusions		
		pEC_{50}	E_{max}% WT)	*n*	pEC_{50}	E_{max} (% WT)	*n*	pEC_{50}	E_{max} (% WT)	*n*	pEC_{50}	E_{max} (% WT)	*n*
C-terminal	CLR/RAMP1	9.47 ± 0.10	100	6	9.19 ± 0.26	79.13 ± 12.58	6	9.08 ± 0.16	75.91 ± 11.09	6	8.29 ± 0.19*	49.42 ± 6.15*	6
	CTR/RAMP1	9.15 ± 0.15	100	4	9.17 ± 0.21	90.24 ± 16.11	4	8.95 ± 0.16	64.64 ± 11.91*	4	8.21 ± 0.18*	30.99 ± 7.59*	4
	CTR	6.67 ± 0.11	100	6	6.46 ± 0.09	115.2 ± 40.88	6	NA	NA	NA	NA	NA	NA
N-terminal	CLR/RAMP1	9.58 ± 0.22	100	3	9.65 ± 0.07	44.24 ± 8.53*	3	9.86 ± 0.10	91.28 ± 24.31	3	9.60 ± 0.16	102.6 ± 3.42	3
	CTR/RAMP1	9.74 ± 0.18	100	4	9.61 ± 0.21	90.76 ± 9.03	3	9.36 ± 0.47	110.8 ± 17.43	3	9.68 ± 0.25	142.4 ± 12.5	4
	CTR	7.46 ± 0.13	100	3	6.93 ± 0.13	53.16 ± 26.38	3	NA	NA	NA	NA	NA	NA

Results are the mean ± s.e.m. of *n* independent experiments performed in duplicate or triplicate. E_{max} values presented here are normalized to the matched WT curve included in each experiment. Values were compared across rows. pEC_{50} values were analyzed by repeated measures ANOVA (groups of four conditions with identical *n* numbers) or a mixed-effects model (groups of four conditions with differing *n* numbers) with post-hoc Holm-Šidák tests, or a paired Student's *t*-test (two conditions with identical *n* numbers), comparing the WT value to each other value. Non-normalized E_{max} values were log-transformed for then analyzed identically to the pEC_{50} values. Statistical significance was accepted when $p < 0.05$, and is indicated by an * (asterisk). E_{max} values presented here are normalized to the WT curve included in each experiment for presentation purposes only. NA – not applicable because we were testing the effect of fusing V_{1-154} to CTR in the absence of RAMP1.

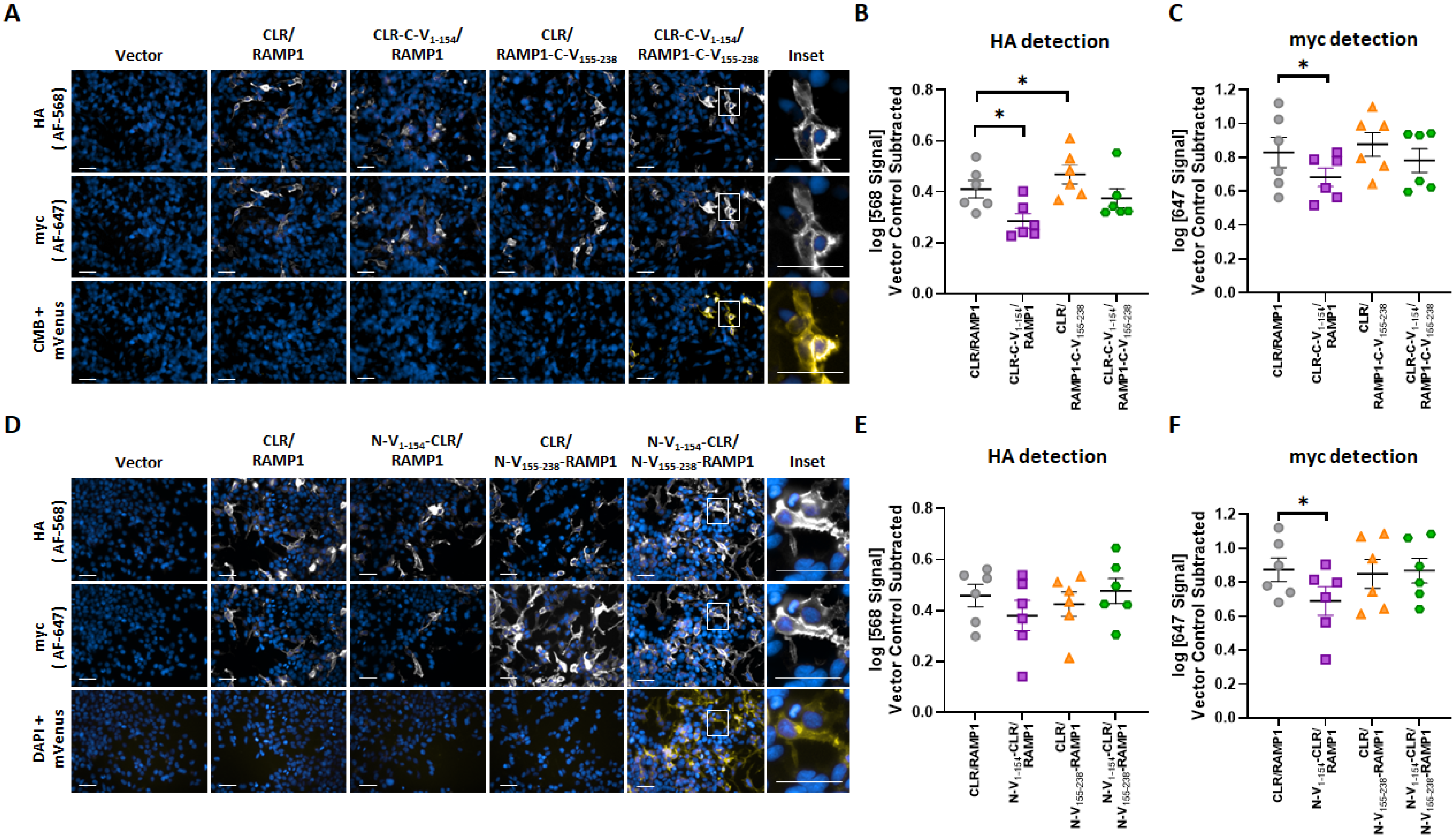

A
Vector
CLR/ RAMP1
CLR-C-V1-154/ RAMP1
CLR/ RAMP1-C-V155-238
CLR-C-V1-154/ RAMP1-C-V155-238
Inset
HA (AF-568)
myc (AF-647)
CMB + mVenus
B
HA detection
log [568 Signal] Vector Control Subtracted
CLR/RAMP1
CLR-C-V1-154/ RAMP1
CLR/ RAMP1-C-V155-238
CLR-C-V1-154/ RAMP1-C-V155-238
C
myc detection
log [647 Signal] Vector Control Subtracted
D
Vector
CLR/ RAMP1
N-V1-154-CLR/ RAMP1
CLR/ N-V155-238-RAMP1
N-V1-154-CLR/ N-V155-238-RAMP1
Inset
HA (AF-568)
myc (AF-647)
DAPI + mVenus
E
HA detection
log [568 Signal] Vector Control Subtracted
F
myc detection
log [647 Signal] Vector Control Subtracted

Supplemental Figure 5. Detection and quantification of cell surface expression and mVenus fluorescence for CLR-based receptor complexes in transfected HEK293S cells. (A-C) are C-terminal fusion constructs, (D-F) are N-terminal fusion constructs. (A, D) Fluorescence results for antibody-based detection of AF-568 and AF-647 (HA-tag and myc-tag, respectively), intrinsic mVenus fluorescence, and a cell marker (CMB or DAPI). AF channels are white, mVenus is yellow, and the cell marker (DAPI or CMB) is blue. Each column within a panel shows the same well and well position. Images are representative of six independent experiments performed in duplicate, triplicate, or quadruplicate. The brightness of images was modified to facilitate visualization on screen and in print and modifications were kept consistent within experiments and channels; care was taken during acquisition so as not to saturate the detector. Scale bars indicate 40 µm. (B, C, E, F) quantified intensity of the HA (AF-568; B, E) or myc (AF-647; C, F) signal within each independent experiment. Each point is an independent experiment; lines indicate mean ± s.e.m. * indicates $p < 0.05$ compared to WT as determined by repeated measures one-way ANOVA with a post-hoc Dunnett's test comparing all conditions to WT.

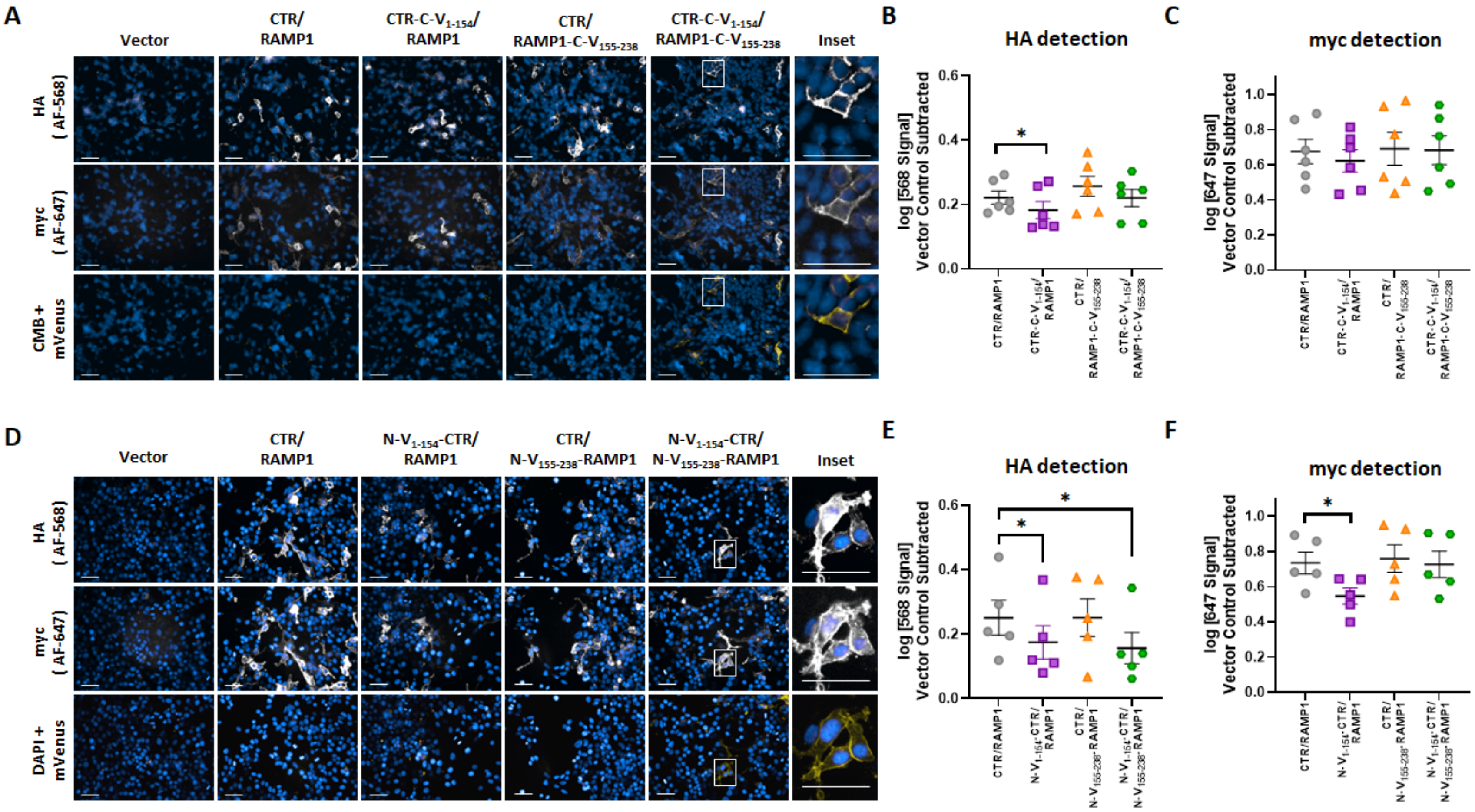
A
Vector
CTR/ RAMP1
CTR-C-V1-154/ RAMP1
CTR/ RAMP1-C-V155-238
CTR-C-V1-154/ RAMP1-C-V155-238
Inset
HA (AF-568)
myc (AF-647)
CMB + mVenus
B
HA detection
log [568 Signal] Vector Control Subtracted
C
myc detection
log [647 Signal] Vector Control Subtracted
D
Vector
CTR/ RAMP1
N-V1-154-CTR/ RAMP1
CTR/ N-V155-238-RAMP1
N-V1-154-CTR/ N-V155-238-RAMP1
Inset
HA (AF-568)
myc (AF-647)
DAPI + mVenus
E
HA detection
log [568 Signal] Vector Control Subtracted
F
myc detection
log [647 Signal] Vector Control Subtracted

Supplemental Figure 6. Detection and quantification of cell surface expression and mVenus fluorescence for CTR-based receptor complexes in transfected HEK293S cells. (A-C) are C-terminal fusion constructs, (D-F) are N-terminal fusion constructs. (A, D) Fluorescence results for antibody-based detection of AF-568 and AF-647 (HA-tag and myc-tag, respectively), intrinsic mVenus fluorescence, and a cell marker (CMB or DAPI). AF channels are white, mVenus is yellow, and the cell marker (DAPI or CMB) is blue. Each column within a panel shows the same well and well position. Images are representative of five or six independent experiments performed in duplicate, triplicate, or quadruplicate. The brightness of images was modified to facilitate visualization on screen and in print and modifications were kept consistent within experiments and channels; care was taken during acquisition so as not to saturate the detector. Scale bars indicate 40 µm. (B, C, E, F) quantified intensity of the HA (AF-568; B, E) or myc (AF-647; C, F) signal within each independent experiment. Each point is an independent experiment; lines indicate mean ± s.e.m. * indicates $p < 0.05$ compared to WT as determined by repeated measures one-way ANOVA with a post-hoc Dunnett's test comparing all conditions to WT.

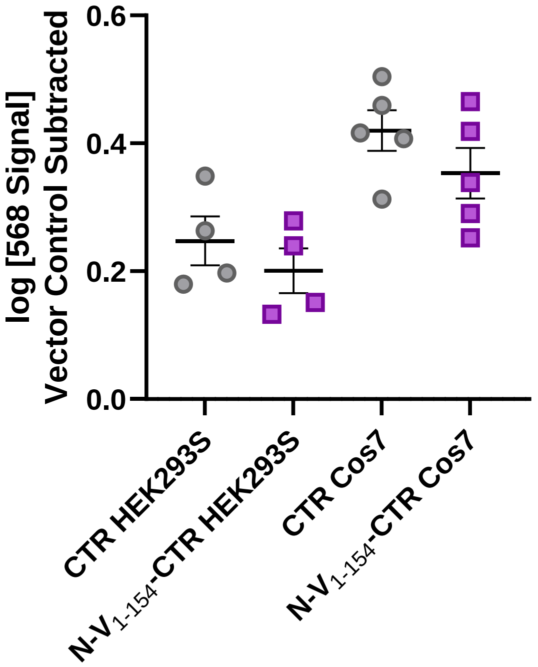

Supplemental Figure 7. The mean intensity of AF-568 in HEK293S and Cos7 cells transiently transfected with either CTR or N-$V_{1\text{-}154}$-CTR. AF-568 corresponds to detection of the N-terminal HA-tag on CTR. Each point is an independent experiment; lines indicate mean ± s.e.m. There was no significant difference between CTR and N-$V_{1\text{-}154}$-CTR in either HEK293S or Cos7 cells as determined by paired Student's *t*-tests comparing 568 intensity between WT and N-$V_{1\text{-}154}$-CTR within cell-types.

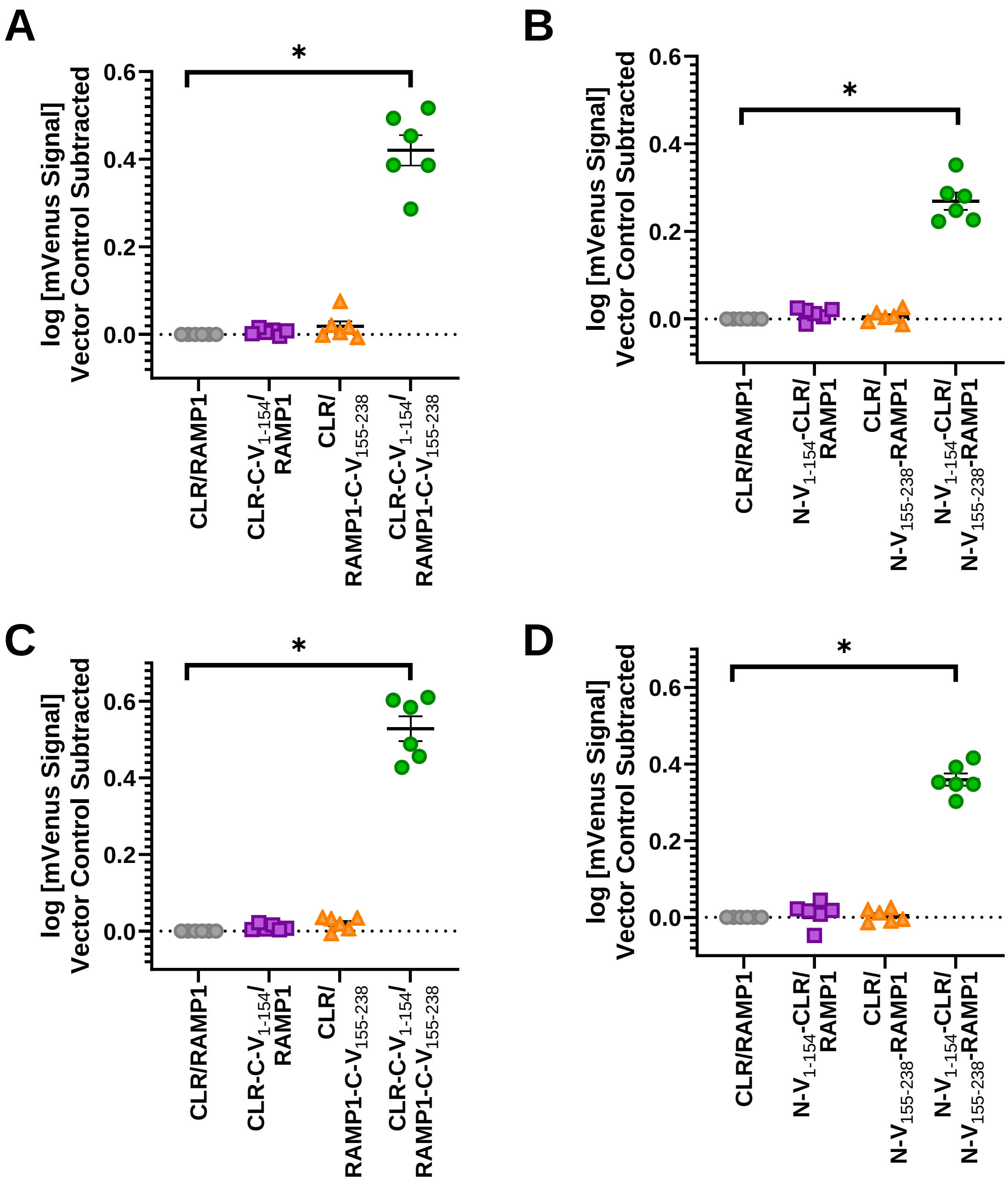

Supplemental Figure 8. Quantification of mVenus signal in cells transiently transfected with CLR/RAMP1. Results are presented for HEK293S (A, B) and Cos7 (C, D) cells with mVenus fragments fused to the C-termini (A, C) or N-termini (B, D) of receptor constructs. Each point is an independent experiment; lines indicate mean ± s.e.m. Data analyzed within panels by one-way ANOVA with post-hoc Dunnett's test, comparing the mVenus intensity in WT transfected cells to the mVenus intensity of each other transfection condition. * indicates a significant ($p < 0.05$) difference relative to WT.

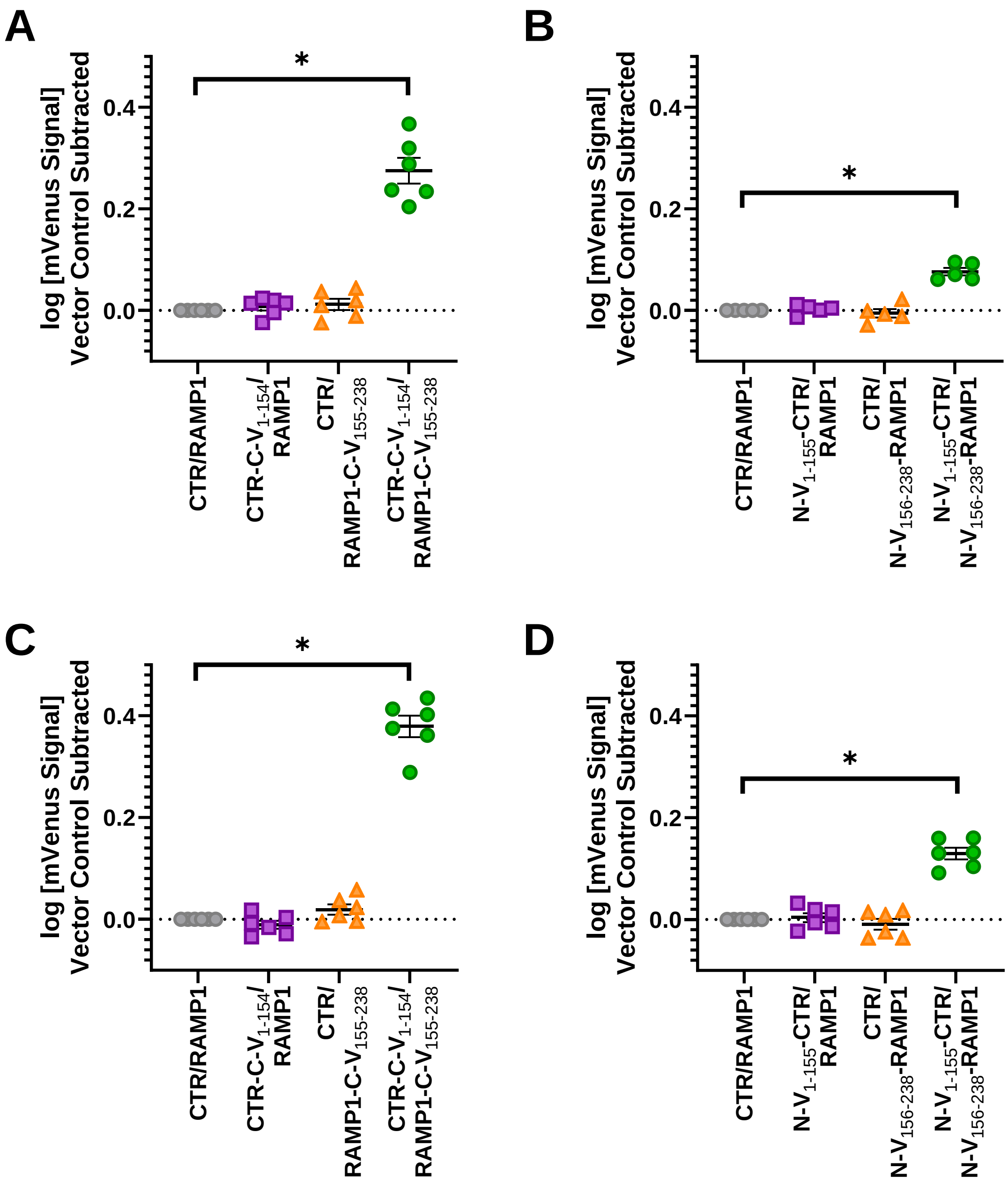

Supplemental Figure 9. Quantification of mVenus signal in cells transiently transfected with CTR/RAMP1. Results are presented for HEK293S (A, B) and Cos7 (C, D) cells with mVenus fragments fused to the C-termini (A, C) or N-termini (B, D) of receptor constructs. Each point is an independent experiment; lines indicate mean ± s.e.m. Data analyzed within panels by one-way ANOVA with post-hoc Dunnett's test, comparing the mVenus intensity in WT transfected cells to the

mVenus intensity of each other transfection condition. * indicates a significant ($p < 0.05$) difference relative to WT.
